# Estimation of mortality hazard ratios by COPD grade using a systematic review and network meta-analysis: deriving grade-, sex-, and age-specific mortality for a German COPD decision model

**DOI:** 10.1038/s41598-026-68641-0

**Published:** 2026-09-09

**Authors:** Tobias Niedermaier, Ulrich Mansmann, Rolf Holle

**Affiliations:** Institute for Medical Information Processing, Biometry and Epidemiology (IBE), Faculty of Medicine, LMU, Marchioninistr. 15, 81377 Munich, Germany

**Keywords:** All-cause mortality, COPD, GOLD grade, Hazard ratios, Network meta-analysis, Diseases, Health care, Medical research, Risk factors

## Abstract

Chronic Obstructive Pulmonary Disease (COPD) imposes substantial economic costs. Cost-effectiveness evaluations in COPD frequently rely on Markov (or other state-transition) models. Their accuracy relies on precise mortality probabilities by COPD grade. The aim of this systematic review was to provide evidence-based hazard ratios (HRs) for grade-specific mortality (GOLD 1–4 at baseline) and to derive grade-, age-, and sex-specific mortality probabilities for patients with COPD in Germany. A systematic PubMed and Europe PMC search was conducted for studies published between 2003 and February 2026. HRs were standardized to the reference group of normal spirometry and pooled by GOLD grade using network meta-analysis. We derived grade-specific mortality probabilities for Germany stratified by 5-year age-groups and sex by combining the pooled HRs with age- and sex-specific German period life-table mortality from the German Federal Statistical Office. Risk of bias was assessed by the Newcastle–Ottawa Scale. In total, 1,459,685 participants from 34 publications were included, with 966,144 having normal spirometry and the others classified into GOLD grades 1–4 at baseline. We found a monotonic increase in mortality with grade, with HRs (95% CIs) of 1.09 (0.97–1.22), 1.57 (1.41–1.75), 2.39 (2.03–2.83), and 4.54 (3.44–6.01) in GOLD 1–4, respectively. Estimated 1-year mortality probabilities for patients with COPD in Germany ranged from 0.15% (females, GOLD 1, ages 45–49) to 64.8% (GOLD 4, males, age ≥ 85). This study provides evidence-based mortality risks for patients with COPD in Germany, stratified by grade, age, and sex, while presenting a transferable framework for deriving similar mortality probabilities in other countries.

**Supplementary Information:**

The online version contains supplementary material available at https://doi.org/10.1038/s41598-026-68641-0.

## Introduction

Chronic Obstructive Pulmonary Disease (COPD) is a chronic lung disease resulting in a continuous (sometimes accelerating) decline in the forced expiratory volume in 1 s (FEV1), i.e. persistent airflow limitation. It is accompanied by chronic inflammation^1^ and frequently by comorbidities such as pulmonary hypertension, cardiovascular diseases, osteoporosis, and lung cancer^2^. Those factors may further impair quality of life and elevate mortality risk. Typical symptoms of COPD include cough, sputum production, dyspnea, and acute exacerbations, which affect quality of life, increase susceptibility to respiratory infections, and may lead to hospitalizations.

Although largely preventable and treatable, COPD remains incurable and continues to represent a major global health challenge. It contributes to chronic illness and premature mortality, both of which impose a substantial and growing economic burden^2^. For example, in Germany, approximately 6% of adults aged 18 years and older have known COPD according to self-administered questionnaires (GEDA 2014/2015-EHIS)^3^, with prevalence rising to 13% among men and 11% among women aged 65 years and older^3^. Tobacco smoking is the leading modifiable risk factor for COPD in Germany^3^ and both smoking cessation and early pharmacological treatment can improve quality of life, reduce exacerbation risk and slow down lung function decline^4^. Key challenges in COPD management in Germany and across Europe include persistent underdiagnosis and delayed diagnosis, together with gaps between guideline recommendations and real-world care, particularly regarding spirometry, inhaler technique/adherence, smoking cessation, vaccination, and pulmonary rehabilitation^2^.

Because COPD progresses slowly, long-term consequences of interventions are typically projected using state-transition (Markov) models^5^. In Germany, Menn et al.^6^ developed a Markov model for COPD in 2012 (see e-Fig. 1), motivating an update of key mortality inputs. As German grade-specific mortality data – particularly for GOLD 3 + 4 – remain limited, we synthesized international cohort evidence and linked pooled hazard ratios (HRs) to German life-table mortality (Destatis). Our objective was to estimate hazard ratios (HRs) for all-cause mortality according to COPD grades, irrespective of the reference group, by synthesizing data from published studies. We focused on the GOLD 2007 classification which is in line with the structure of the Markov model. Additionally, we applied these HRs to calculate grade-, age-, and sex-specific mortality probabilities for patients with COPD, using Germany as a use case.

**Fig. 1:**
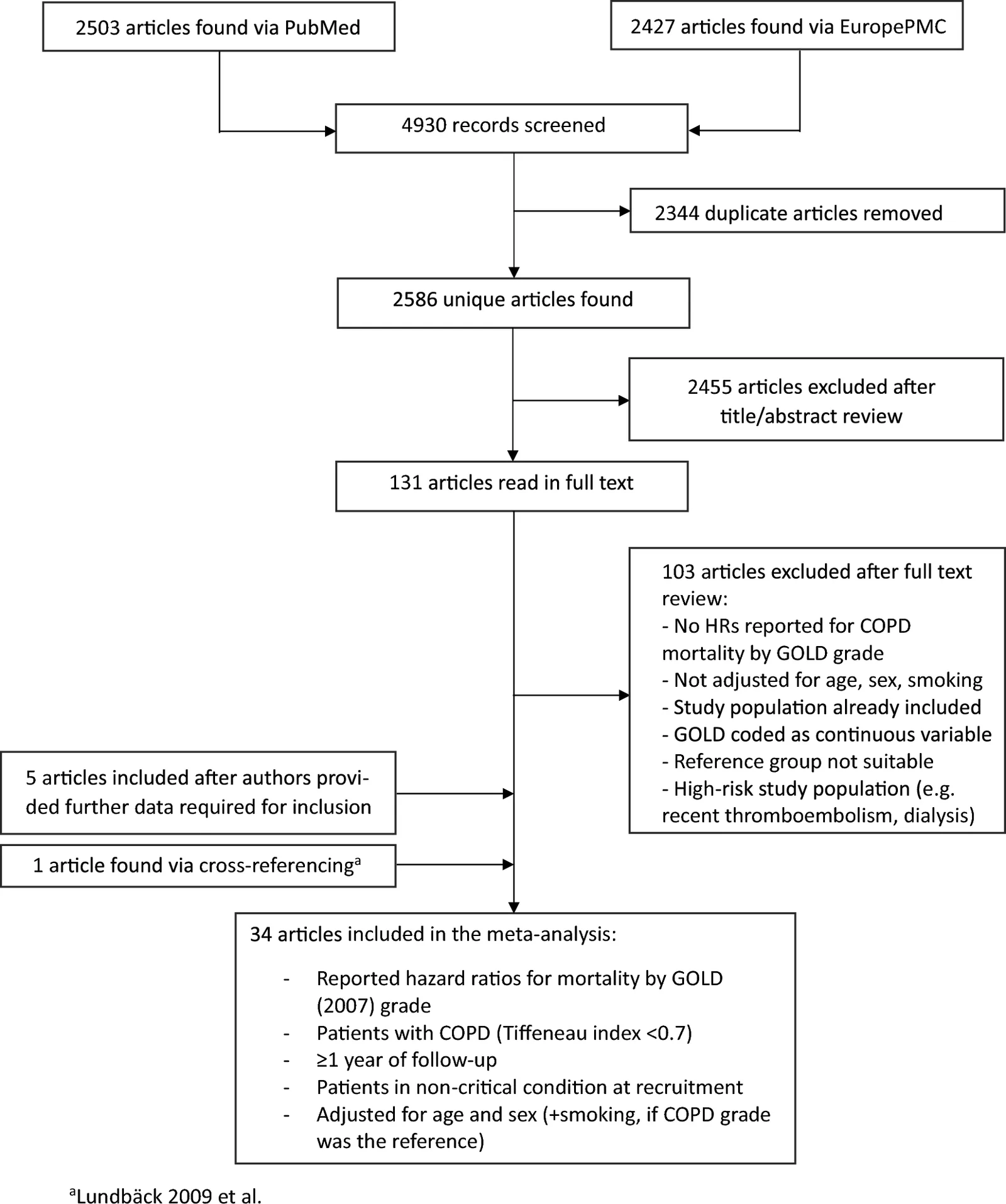
Flow diagram of study selection.

## Material and methods

We first conducted a systematic literature review of longitudinal studies reporting mortality by COPD grade. From eligible studies, basic characteristics such as sample size, setting, and follow-up duration were extracted. Next, HRs of all-cause mortality for different COPD grades were extracted, along with information on the reference group and covariate adjustment. Finally, we linked the pooled HRs to age- and sex-specific German life-table mortality to derive grade-specific annual mortality risks for use in the decision model. We followed the Preferred Reporting Items for Systematic reviews and Meta-Analyses (PRISMA) 2020 guidelines^7^ when conducting this review. A versioned review protocol (reflecting the final methods as implemented) has been made publicly available on OSF (https://osf.io/zwky4/overview?view_only=c3712d3a845740eca968ccdaefc5c5ae).

### Classification of COPD severity

In our literature search, we focused on studies following the Global Initiative for Chronic Obstructive Lung Disease (GOLD) classification from 2007 for disease severity because this classification is the basis of our^6^ and other published Markov models of COPD. The classification is based on measurements of forced expiratory volume in 1 s (FEV1) and of Forced Vital Capacity (FVC). Thus, COPD was defined by airflow obstruction (FEV1/FVC < 0.70), and severity grades were based on percent-predicted FEV1 cut-offs (GOLD 1: ≥ 80%, GOLD 2: 50–79%, GOLD 3: 30–49%, GOLD 4: < 30%)^8^.

### Literature search and study selection

We conducted a systematic literature review using PubMed and EuropePMC to identify relevant literature (last search: February 10, 2026). Our aim was to summarize data on HRs for all-cause mortality for the individual GOLD grades (1–4) irrespective of the reference group.

The search terms included “COPD”, “mortality”, terms for longitudinal studies, terms for disease grade, and for pertinent outcomes. In addition, references from cited and citing articles were checked for further potentially relevant articles. Details on the search terms and the data extraction process are shown in the e-Appendix “PubMed search”.

We included longitudinal studies published up to February 2026 with ≥ 12 months of follow-up. This threshold was chosen because cohorts often recruit participants who are clinically stable at baseline, which may result in an early period with comparatively low observed mortality and could underestimate mortality in studies with short follow-up. GOLD grade was defined at baseline (spirometry at study entry) and treated as a baseline exposure in the main analysis (i.e., not modeled as time-varying).

Eligible studies were original peer-reviewed articles reporting adjusted HRs and its 95% confidence intervals (CIs) for all-cause mortality in individuals with COPD at GOLD grades 1–4. Acceptable reference categories were normal spirometry or any individual GOLD grade (1–4). We required hazard ratios to be adjusted at least for age and sex, and for smoking exposure (smoking status and/or pack-years), because these are core confounders of the association between lung function impairment and mortality. Additional adjustment factors varied by study (e.g., BMI, education/socioeconomic status, comorbidities, and lifestyle factors) and are summarized in Table 2.

To derive age- and sex-specific mortality probabilities for Germany, we used age- and sex-specific German applied the pooled grade-specific hazard ratios to sex- and age-stratified baseline mortality. This implicitly assumes no effect modification of the hazard ratios by age or sex (i.e., proportional hazards across strata), which we acknowledge as a modeling assumption. We additionally required adjustment for smoking exposure when normal spirometry was the reference group. When a COPD grade was used as the reference category, lack of smoking adjustment was considered acceptable because the comparison is within obstructed individuals (although residual confounding cannot be excluded). Studies on mortality only in hospitalized patients (hospital cohorts), conference abstracts, and unpublished manuscripts were not eligible. When necessary, we contacted study authors to obtain missing stratified HRs (i.e., HRs by GOLD grade) and integrated the received data into the meta-analysis.

Normal spirometry was defined as the absence of airflow obstruction (FEV1/FVC ≥ 0.70) with preserved lung function, i.e. FEV_1_ ≥ 80% of the predicted normal value (“percent predicted”) where available. Because operational definitions varied across cohorts, we also included studies that defined the reference group more broadly (e.g., no airflow obstruction and/or no respiratory symptoms), and documented the study-specific definition during extraction. If HRs for several models with varying degree of covariate adjustment were reported, we extracted data from the model with the most comprehensive confounder adjustment, unless the model adjusted for covariates lying on the causal pathway between COPD grade and mortality (e.g., severity of symptoms). In that case, we used estimates from a model with a more eligible selection of covariates. Specifically, physical activity or COPD medication may represent over-adjustment if affected by COPD severity. Thus, we conducted sensitivity analyses excluding studies that adjusted for these variables.

We excluded studies that reported mortality outcomes only for selected high-risk populations (e.g., hospitalized patients, recently discharged individuals, or patients receiving dialysis). We also excluded studies using GOLD grade 0 (FEV₁/FVC ≥ 0.7) as the reference category, because this group is not sufficiently homogeneous or comparable. That group may comprise individuals with normal spirometry as well as those with preserved ratio, impaired spirometry (PRISm). Studies fulfilling all eligibility criteria were pooled in a network meta-analysis.

### Risk of bias assessment

We used the Newcastle–Ottawa Scale (NOS) to assess the risk of bias in the individual studies. The NOS comprises 8 questions across 3 categories (4 for selection, 1 for comparability, 3 for outcome) which we translated into “good”, “fair”, and “poor” quality. Evaluations were done by one researcher (T.N.) and ambiguities and discrepancies were resolved in consensus with a second researcher (R.H.). As an auxiliary quality control step, extracted datasets were screened using a large language model (ChatGPT 5.2 Thinking, OpenAI) to identify potential inconsistencies or formatting errors in extracted data, eligibility/exclusion decisions, and Newcastle-Ottawa Scale risk-of-bias assessments. The model was not used as an independent reviewer and had no decision-making role. All outputs were treated only as prompts for human re-checking; suggested changes were accepted only after manual verification against the original publications, and ambiguous cases were discussed with a second author. Further details are provided in the Supplement. The AI output was used solely for error detection support and did not replace independent human review. We did not apply GRADE because the study synthesizes prognostic associations from observational cohorts within a network meta-analysis, for which several standard GRADE domains are not readily operationalized.

### Statistical methods

The main comparison was all-cause mortality in any GOLD grade vs. any reference groups (normal spirometry, or any individual GOLD grade). Effect estimates, reference groups, and covariates adjusted for were tabulated. We collected information on the study name along with hazard ratios (HRs) and 95% confidence intervals, exposure and reference group in a data extraction sheet. For pooling, the natural logarithm of the HRs was calculated, and the corresponding standard errors were computed as (log(upper ci) – log(lower ci))/(2*1.96). For Ogata et al.^43^, who reported Bonferroni-adjusted 99.17% confidence intervals, standard errors were reconstructed using the corresponding 99.17% normal quantile instead of 1.96. To achieve consistency in reference groups, network meta-analysis was employed. In the network meta-analysis, all hazard ratios are re-expressed on a common scale (normal spirometry as reference) and then pooled. Weighted least squares and study-clustered standard errors were used to address the fact that each study (typically) contributed several estimates. We used a random effects model to account for heterogeneity among the studies, caused e.g. by differences in follow-up durations, covariate adjustments, healthcare systems, smoking prevalence, etc. Statistical heterogeneity was assessed by the I^2^ statistic (based on Cochran’s Q) which ranges between 0 and 100%. We explored potentially influential studies using leave-one-out sensitivity analyses. We assessed small-study effects (publication bias) primarily by visual inspection of funnel plots and complemented this with tests for funnel-plot asymmetry (Begg’s, Egger’s, and Thompson-Sharp test). We stratified studies into clinical studies and population studies because we suspected the latter to be on average healthier (e.g. with fewer comorbidities) even within the same grade. In additional subgroup analyses, we stratified studies according to the median of all reported (mean or median) follow-up durations. The potential influence of follow-up duration was also assessed by stratification (dichotomized at median of mean follow-up durations). Inconsistency was further investigated via netheat plots^9^. To derive sex- and age-stratified COPD mortality risks for Germany, we used age- and sex-specific German period life tables from the German Federal Statistical Office (Destatis). In the main analysis, baseline mortality was taken from the 2022/24 German period life tables^10^. These life tables provide mortality probabilities for age intervals, which were converted to annual baseline hazards as $h_0 = -\frac{\ln(1 - _nq_x)}{n}$, where $_n q_x$ denotes the life-table probability of dying within an age interval of length n. Hazards were calculated separately for men and women and for 5-year age groups starting at age 45. For the open age group 85 years and older, hazards were aggregated from the 85–89, 90–94, 95–99, and 100+ life-table components using person-years weights^11^.

Under proportional hazards, grade-specific hazards were calculated as $h_v = HR_v \cdot h_0$, where $HR_v$ denotes the pooled hazard ratio for GOLD grade v versus normal spirometry. These hazards were converted back to 1-year mortality probabilities as $p_v = 1-exp(-HR_v\cdot h_0).$ For readability, mortality probabilities are reported as expected deaths per 100,000 persons over one year. As sensitivity analyses, we repeated the calculation using the German 2023/25 and 2017/19 period life tables, applying the same calculation procedure as in the main analysis. Mortality probabilities provide an approximation to the 1-year all-cause mortality risk of the reference group normal spirometry assuming COPD prevalence is modest relative to the total population, where HR_v_ denotes the pooled hazard ratio for GOLD grade *v* versus normal spirometry.

The mapping of grade-specific hazards to 1-year mortality probabilities is described in detail in the Supplement. This mapping is non-linear because HRs act multiplicatively on hazards (and cumulative hazards), corresponding to a power transformation of survival probabilities. All analyses were carried out in R (version 4.5.3). Network meta-analyses were implemented using the netmeta package.

## Results

### Literature search

The literature search identified 34 relevant articles in total^12–45^. Of those, 19 reported HRs (excluding reference) for GOLD grade 1^12–15,18,19,22,23,25,26,29–33,35,39,41,43^. For GOLD grades 2 and 3, 26^12–16,18,19,21–24,28–30–36,38–45^ and 22^13,15–17,20–24,27–30,34,36–40,42,43,45^ studies were included in the analysis, respectively. For GOLD grade 4, 20^13,16,17,20–22,24,27–30,34,36–40,42,44,45^ studies were considered in the analysis. Articles were mainly excluded because they reported HRs for combined GOLD grades (e.g. II-IV) or because the HRs were not adjusted for age and sex. The flow diagram of included studies is shown in Fig. 1.

### Study characteristics

We primarily focused on studies reporting on mortality according to post-bronchodilator (BD) FEV1. However, we also included studies that conducted spirometry without BD (see e-Table 1) because FEV1 is not fully reversible by BD in patients with COPD^46^. Study characteristics are summarized in Table 1. Overall, 1,459,685 participants were included in the study: 134,030 participants classified with COPD grade GOLD 1, 239,564 participants with GOLD 2, 94,416 participants with GOLD 3, 25,531 participants with GOLD 4, and 966,144 participants with normal spirometry. Cohort contributions were dominated by Whittaker et al.^36^ for GOLD 1–4, and by Ke et al.^39^ for the normal-spirometry reference group. The follow-up period across the studies varied between 2.2^24^ and 30^22^ years. The included studies were published between 2003 and 2026. Most studies were of very good quality (partly as a result of our in-/exclusion criteria), with a median of 8 out of 9 points on the NOS (e-Table 1). Reasons for exclusion of ineligible studies (despite reporting risk estimates for mortality by GOLD grade) are listed in e-Table 2. For included studies, Table 2 provides the extracted HRs by grade, along with the corresponding reference categories and covariate adjustment sets.

**Table 1:** Characteristics of all studies included in the analysis.

First author, year, reference	Study Location (study name)	Sample size					Follow-up ^b^	Setting ^d^
		GOLD 1	GOLD 2	GOLD 3	GOLD 4	Normal		
Mannino 2003^12^	USA (NHANES I 1971–1975)	213	247	48 (3 + 4)		1,097	17.9^**c**^	p
Ekberg-Aronsson 2005^13^	Sweden (screening)	1,779^**a**^	1,326^**a**^	197^**a**^	54^**a**^	15,921	21.5	p
Mannino 2006^14^	USA (ARIC)	1,679	1,484	271 (3 + 4)		8,661	9.0	p
Stavem 2006^15^	Norway (screening)	142	107	16	NR	1,223	26	p
Lundbäck 2009^16^	Sweden (OLIN)	96	134	24	12	NR	≤ 20	p
Alfageme 2010^17^	Spain (clinical cohort)	NR	195	287	114	NR	2.7	c
Mannino 2011^18^	USA (LHS)	1,565	2,540	19 (3 + 4)		1,058	12.8^**c**^	p
Mannino 2012^19^	USA (NHANES III)	1,081	826	245 (3 + 4)		3,299	≤ 18	p
Johannessen 2013^20^	Norway (GenKOLS)	NR	510	272	130	NR	6.4	c
Leivseth 2013^21^	Norway (HUNT2)	424	883	204	29	NR	14.6^**c**^	c
Mattila 2015^22^	Finland (screening)	165	213	72	13	6,173	30	p
Brusse-Keizer 2017^23^	Netherlands (COMIC)	58	283	263	61	NR	≥ 3	c
Gedebjerg 2018^24^ ^e^	Denmark (database linkage)	1,661	11,772	12,899	7,433	NR	2.2^**c**^	c
Perez-Padilla 2018^25^	Chile, Brazil, Uruguay (PLATINO)	323	201 (2–4)			942	≤ 9	p
Wijnant 2020^26^	Netherlands (Rotterdam study)	419	493 (2–4)			4,150	≤ 9.3	p
Brat 2021^27^	Czech Republic (CMRD)	NR	266	361	93	NR	≤ 6	c
Damkjaer 2021^28^	Denmark (clinic cohort)	39	332	392	186	NR	2.8^**c**^	c
Guo 2021^29^	Taiwan (Taiwan MJ cohort)	1,520	8,291	3,733	1,176	207,199	16.2	p
Hasenstab 2021^30^	USA (COPDGene)	695	1,723	1,022	529	3,855	10	p
He 2021^31^	UK (ELSA)	721	1,099 (2–4)			3,450	7.7	p
Washio 2022^32^	Japan (Hisayama Study)	216	307 (2–4)			2,208	5.3^**c**^	p
Kaaks 2022^33^	Germany (LUSI)	92	236	41 (3 + 4)		1,307	12.1^**c**^	p
Cheng 2023^34^	PR China (RealDTC participants)	266	1,035	856	342	NR	3.2^**c**^	c
Cadham 2024^35^	USA (NHANES I 2007–2012)	810	836	143 (3 + 4)		9,677	9.4^**c**^	p
Whittaker 2024^36^	England (database linkage)	69,476	133,624	46,556	8,382	NR	5.5	c
Egerod 2025^37^ ^e^	Netherlands (Groningen Severe COPD Cohort)	NR	NR	386	559	NR	3.0	c
Hyung 2025^38^	Korea (KOCOSS)	510	1,037	395	47	NR	5.0^**c**^	c
Ke 2025^39^	China (China Kadoorie Biobank)	4,945	13,127	5,596	1,448	341,975	16.0^**c**^	p
Lin 2025^40^ ^e^	Taiwan (Taiwan National Health Insurance claim database)	8,846	18,520	9,254	2,308	NR	4	p
Ma 2025^41^	UK (UK Biobank study)	31,587	18,171	2,274 (3 + 4)		302,540	11.7	p
Niedermaier 2025^42^	Germany (COSYCONET)	405	1,139	889	252	NR	≤ 6	c
Ogata 2025^43^	Japan (Fukuoka Tobacco-Related Lung Disease registry study group)	57	137	109	67	NR	5.0^**c**^	c
Wallström 2025^44^ ^e^	Sweden (Sw. National Airway Register)	3,795	21,187	10,766	2,262	NR	3.7^**c**^	c
Casanova 2026^45^ ^e^	Spain (CHAIN study)	73	209	154	33	NR	10	c

Abbreviations: ARIC = Atherosclerosis Risk in Communities. CMRD = Czech Multicenter Research Database. COMIC = Cohort of Mortality and Inflammation in COPD; GenKOLS = Genetics of Chronic Obstructive Lung Disease study. GOLD = Global Initiative for Chronic Obstructive Pulmonary Disease. HUNT = Helseundersøkelsen i Nord-Trøndelag; LHS = Lung Health Study; LUSI = German Lung Cancer Screening Intervention Study; NHANES = National Health and Nutrition Examination Survey; NR = not reported / no cases; OLIN = Obstructive Lung Disease in Northern Sweden; PLATINO = Proyecto LatinoAmericano de Investigación en Obstrucción Pulmonar; Taiwan MJ cohort = screening program for Taiwan residents provided by the MJ Health Management Institution.

^a^ Combined sample size (males and females).

^b^ Average (mean) follow-up time for mortality in years, unless stated otherwise.

^c^ Median follow-up time for mortality in years.

^d^ population vs. clinical setting.

^e^ Additional data necessary for inclusion obtained from corresponding author.

Effective sample size per grade is smaller in Gedebjerg et al. 2018 because estimates are based on 23,007 patients out of 33,765 recruited patients.

**Table 2:** Adjusted hazard ratios (95% CIs).

First author, year, ref	GOLD 1	GOLD 2	GOLD 3	GOLD 4	Reference	Covariate adjustment
Mannino 2003^12^	1.2 (1.01, 1.4)	1.6 (1.4, 2.0)	2.7 (2.1, 3.5)		Normal	Age, sex, race, education, smoking (status + pack- years), years since regularly smoked, BMI
Ekberg-Aronsson 2005^a^^13^	1.12 (1.01, 1.25)	1.46 (1.31, 1.62)	2.88 (2.34, 3.54)	4.34 (3.00, 6.28)	Normal	Age, sex, smoking status
Mannino 2006^14^	1.40 (1.10, 1.60)	2.40 (2.00, 2.90)	5.7 (4.4, 7.3)		Normal	Age, sex, smoking status, BMI, race, pack-years of smoking, years since last smoked, educational level
Stavem 2006^15^	1.30 (1.00, 1.69)	1.77 (1.35, 2.32)	1.81 (0.96, 3.42)	NR	Normal	Age, smoking status, physical fitness, BMI, systolic BP, serum cholesterol (Note: only men included.)
Lundbäck 2009^16^	ref.	1.52 (1.03, 2.26)	2.53 (1.44–4.44)	7.29 (3.39, 15.65)	GOLD 1	Age, sex, smoking, ischaemic + other heart disease, family history of OAD, socio-economic status
Alfageme 2010^17^	NR	ref.	NR	2.38 (1.61, 3.53)	GOLD 2	Age, current smoking, cardiopathy, neoplasia, acute exacerbations (highest quartile)
Mannino 2011^18^	1.06 (0.80, 1.40)	1.54 (1.20, 1.98)	1.45 (0.41, 5.15)		Normal	Age, sex, race, education, smoking, BMI, randomization cohort
Mannino 2012^19^	1.15 (0.98, 1.35)	1.78 (1.51, 2.10)	3.29 (2.50, 4.33)		Normal	Age, sex, race, BMI, smoking, diabetes, CVD, education, poverty income ratio
Johannessen 2013^20^	NR	ref.	1.7 (1.3, 2.2)	3.7 (2.7, 5.0)	GOLD 2	Age, sex, smoking, BMI, comorbidities
Leivseth 2013 ^a^^21^	ref.	1.68 (1.39, 2.01)	2.87 (2.29, 3.60)	4.85 (3.16, 7.44)	GOLD 1	Age, smoking, education + stratification by sex
Mattila 2015^22^	1.27 (1.06, 1.51)	1.40 (1.21, 1.63)	1.55 (1.21, 1.97)	2.85 (1.65, 4.94)	Normal	Age, sex, smoking
Brusse-Keizer 2017^23^	4.6 (1.9, 11.1) (4 vs. 1)	4.5 (2.6, 7.6) (4 vs. 2)	1.8 (1.2, 2.9) (4 vs. 3)	NR	GOLD 1,2,3	Age, sex, BMI, myocardial infarction, congestive heart failure, diabetes
	2.5 (1.1, 5.6) (3 vs. 1)	2.5 (1.6, 3.7) (3 vs. 2)	NR	NR	GOLD 1 + 2	
Gedebjerg 2018^24^^d^	ref.	1.37 (1.18, 1.59)	2.46 (2.13, 2.84)	4.56 (3.94, 5.29)	GOLD 1	Age, sex, marital status
Perez-Padilla 2018^25^	1.53 (1.01, 2.30)	2.95 (1.90, 4.50)			Normal	Age, sex, smoking (pack- years and cigarettes/day), BMI, education, comorbidities
Wijnant 2020^26^	1.00 (0.70, 1.30)	NR	NR	NR	Normal	Age, sex, current smoking and pack-years, BMI
Brat 2021^27^	NR	ref.	1.61 (1.24, 2.10)	2.76 (1.96, 3.88)	GOLD 2	Age and sex
Damkjaer 2021^28^	ref.	1.54 (0.45, 5.33)	2.31 (0.68, 7.87)	4.07 (1.18, 14.1)	GOLD 1	Age, sex, smoking
Guo 2021^b^^29^	1.16 (1.00, 1.35)	1.35 (1.28, 1.43)	1.70 (1.59, 1.81)	1.99 (1.81, 2.19)	Normal	Age, sex, cigarette smoking, city, education, BMI, PM2.5, occupational exposure, calendar season, year, alcohol, physical activity, fruit + vegetable intake
Hasenstab 2021^30^	0.83 (0.64, 1.06)	1.51 (1.29, 1.78)	2.80 (2.38, 3.30)	7.15 (6.03, 8.46)	Normal	Sex, race, smoking (status + pack-years), age as underlying time scale
He 2021^31^	1.45 (1.15; 1.83)	1.85 (1.55; 2.20)			Normal	Age, sex, marital status, education, BMI, baseline CVD + cancer, smoking + drinking status, physical activity
Washio 2022^32^	1.02 (0.55, 1.87)	NR	NR	NR	Normal	Age, sex, current smoking, smoking pack- years, BMI
Kaaks 2022^33^	1.40 (0.77, 2.55)	1.74 (1.22, 2.49)	4.33 (2.68, 6.99)		Normal	Age, sex, lifetime smoking duration, cigarettes/day, time since quitting (ex-smokers), diabetes
Cheng 2023^34^	ref.	1.01 (0.53, 1.93)	1.19 (0.62, 2.28)	1.91 (0.96, 3.82)	GOLD 1	Age, sex, BMI, smoking
Cadham 2024^35^	1.2 (0.9–1.6)	2.1 (1.7, 2.6)	4.2 (2.7, 6.5)		Normal	Age, sex, BMI, smoking status, race/ethnicity
Whittaker 2024^36^	ref.	1.45 (1.42, 1.48)	2.33 (2.28, 2.39)	4.10 (3.95, 4.25)	GOLD 1	Age, sex, smoking status, all study baseline characteristics
Egerod 2025^37^ ^d^	NR	NR	ref.	2.634 (1.753; 3.960)	GOLD 3	Age, sex, smoking status, pack-years
Hyung 2025^38^	ref.	1.45 (1.01, 2.07)	2.41 (1.64, 3.54)	4.65 (2.53, 8.55)	GOLD 1	Age, sex, BMI, smoking, asthma, CVD, treatments
Ke 2025^39^	1.07 (1.01, 1.14)	1.33 (1.29, 1.37)	2.27 (2.18, 2.35)	4.16 (3.91, 4.43)	Normal	Age, sex, study area, education, occupation, household income, marital status, alcohol, smoking status (active + passive), physical activity, cooking, heating fuel usage, intake of fruits, vegetables, meat, obesity (general + abdominal)
Lin 2025^40^ ^d^	ref.	1.217 (1.125–1.316)	1.744 (1.604–1.897)	2.686 (2.407–2.997)	GOLD 1	Age, gender, body height, weight, and race
Ma 2025^41^ ^a^	1.15 (1.10, 1.20)	1.61 (1.54, 1.69)	2.90 (2.66, 3.17)		Normal	Age, sex, Townsend Deprivation Index, BMI, education, race, smoking status, alcohol consumption
Niedermaier 2025^42^^c^	ref.	1.83 (1.13–2.97)	3.91 (2.42–6.34)	11.79 (6.92–20.12)	GOLD 1	Age, sex, smoking status
Ogata 2025^43^ ^d^	0.13 (0.02–0.77)	0.12 (0.03, 0.41)	0.34 (0.14, 0.83)	ref.	GOLD 4	Age, sex, BMI, smoking, ICS, LABA/LAMA, history of asthma, CRP
Wallström 2025^44^ ^e^	ref.	1.43 (1.31–1.56)	2.59 (2.36–2.83)	5.28 (4.74–5.87)	GOLD 1	Age, sex, smoking, BMI, baseline CVD, baseline cardioprotective medication
Casanova 2026^45^ ^e^	ref.	1.20 (0.49–2.94)	3.09 (1.30–7.36)	3.95 (1.42–10.99)	GOLD 1	Age, sex, smoking, BMI

Abbreviations: CI = confidence interval; CRP = C-reactive protein; CVD = cardiovascular disease; GOLD = Global Initiative for Chronic Obstructive Pulmonary Disease; ICS = inhaled corticosteroids; LABA = long-acting beta-adrenoceptor agonist; LAMA = long-acting muscarinic agonists; NA = not applicable; normal = normal spirometry; NR, not reported; OAD = obstructive airway disease (asthma, COPD, chronic bronchitis, emphysema); ref. = reference group.

^a^Estimates from Ekberg-Aronsson et al., Leivseth et al., and Ma et al. were pooled with a fixed effects model from hazard ratios that were reported stratified by another variable using the same reference.

^b^Estimates from Guo et al. 2021 were taken from the Supplement (Table E2, “Sensitivity 6”) because that was the only model in which only baseline measurements were considered, i.e. (like all other studies) GOLD grade and other variables were not modeled as time-varying.

^c^Estimates from Niedermaier et al. 2025 were taken from the Supplement (Supplementary Table 1) because those estimates were adjusted for smoking.

^d^The confidence intervals reported by Ogata et al. 2025 were Bonferroni-adjusted 99.17% confidence intervals. Accordingly, standard errors for these estimates were reconstructed using the corresponding 99.17% confidence level.

^e^Additional data necessary for inclusion obtained from corresponding author.

### COPD severity and all-cause mortality

Table 3 shows the pooled HRs from network meta-analysis, conducted using a random effects model. In the main analysis, the network meta-analysis resulted in HRs (95% CIs) versus normal spirometry of 1.09 (0.97–1.22) for GOLD 1, 1.57 (1.41–1.75) for GOLD 2, 2.39 (2.03–2.83) for GOLD 3, and a HR of 4.54 (3.44–6.01) for GOLD 4. In population studies, HRs compared to normal spirometry gradually increased from 1.11 (1.01–1.22) over 1.63 (1.48–1.81), 2.30 (1.76–2.99), and 4.06 (2.01–8.19) for GOLD 1–4, respectively. In clinical settings, HRs for GOLD 2, 3, and 4 vs. GOLD 1 were 1.40 (1.20–1.63), 2.28 (1.85–2.81), and 4.46 (3.29–6.04), respectively. When setting the reference group for population studies to GOLD 1, HRs for GOLD 2–4 in population studies were very similar to those from clinical studies. All associations were slightly weaker in studies with ≥ 9 years of longer follow-up, ranging from 1.10 (0.98–1.23) (GOLD 1) to 3.93 (1.94–7.97) (GOLD 4). When leaving out one study at a time, only the study by Guo et al. showed some impact on the pooled estimates (e-Table 3). In a further sensitivity analysis, we included the most recent publications for the HUNT (Bhatta 2020 instead of Leivseth 2013) and the NHANES III study (Zou 2025 instead of Mannino 2012) respectively. Results did not differ notably, though. Furthermore, exclusion of studies that adjusted for physical activity or medication at baseline changed the pooled estimates only slightly, without altering the overall pattern of association (e-Table 4). Heterogeneity was explored quantitatively through subgroup analyses by study setting and follow-up duration; however, I² remained high across most strata.

**Table 3:** Results of network meta-analysis.

Grade	Pooled hazard ratio (95% CI)			
	All studies^1^	Population studies^a^	Clinical setting^b^	Population studies^b^
Weighted least squares + study-clustered standard errors				
Normal	Ref	Ref	NA	0.90 (0.82–0.99)
GOLD 1	1.09 (0.97–1.22)	1.11 (1.01–1.22)	Ref	Ref
GOLD 2	1.57 (1.41–1.75)	1.63 (1.48–1.81)	1.40 (1.20–1.63)	1.47 (1.32–1.65)
GOLD 3	2.39 (2.03–2.83)	2.30 (1.76–2.99)	2.28 (1.85–2.81)	2.07 (1.55–2.75)
GOLD 4	4.54 (3.44–6.01)	4.06 (2.01–8.19)	16 (3.29–6.04)	3.65 (1.96–6.81)
Heterogeneity (I^2^)	89.9%	91.1%	85.6%	91.1%
Stratified by follow-up duration^c^				
Less than 9 years				
GOLD 1	1.33 (0.11–16.68)	NA^4^	NA^4^	NA^4^
GOLD 2	1.78 (0.49–6.47)	NA^4^	NA^4^	NA^4^
GOLD 3	2.85 (0.77–10.50)	NA^4^	NA^4^	NA^4^
GOLD 4	5.63 (1.33–23.79)	NA^4^	NA^4^	NA^4^
Heterogeneity (I^2^)	88.3%			
9 years or more				
GOLD 1	1.10 (0.98–1.23)	NA^4^	NA^4^	NA^4^
GOLD 2	1.62 (1.45–1.81)	NA^4^	NA^4^	NA^4^
GOLD 3	2.29 (1.75–3.01)	NA^4^	NA^4^	NA^4^
GOLD 4	3.93 (1.94–7.97)	NA^4^	NA^4^	NA^4^
Heterogeneity (I^2^)	92.0%			

Abbreviations: CI = confidence interval; GOLD = Global Initiative for Chronic Obstructive Pulmonary Disease; normal = normal spirometry; ref. = reference group. ^a^Reference: normal spirometry. ^b^Reference: GOLD 1. ^c^Dichotomized at the median of reported follow-up durations among studies reporting mean or median follow-up. ^4^Insufficient number of studies to stratify by setting and follow-up duration simultaneously.

This table presents the pooled hazard ratios (95% CIs) with reference group normal (for all studies and population studies) by GOLD grade and with reference group 1 (for clinical studies), obtained from network meta-analysis, conducted using a random effects model. All included studies were considered in the analyses regardless of their reference group.

**Table 4:** A 1-year mortality risks (95% CI) per 100,000 individuals in Germany, stratified by sex, age group, and GOLD grade.

Age group	Sex	GOLD 1	GOLD 2	GOLD 3	GOLD 4
45 – 49 years	Male	262 (233–293)	377 (339–419)	574 (486–678)	1087 (823–1435)
	Female	146 (130–164)	211 (190–234)	321 (272–379)	608 (460–804)
50 – 54 years	Male	437 (390–489)	629 (566–699)	957 (811–1129)	1809 (1371–2385)
	Female	242 (216–271)	349 (314–388)	531 (450–627)	1006 (762–1328)
55 – 59 years	Male	733 (654–822)	1056 (950–1173)	1604 (1360–1892)	3024 (2295–3979)
	Female	398 (355–446)	573 (515–637)	871 (738–1029)	1648 (1249–2174)
60- 64 years	Male	1260 (1124–1411)	1812 (1631–2012)	2747 (2330–3237)	5151 (3921–6755)
	Female	677 (604–758)	974 (877–1083)	1481 (1255–1747)	2793 (2119–3676)
65 – 69 years	Male	2014 (1799–2255)	2891 (2604–3210)	4372 (3714–5144)	8138 (6218–10,617)
	Female	1100 (982–1233)	1583 (1424–1758)	2401 (2036–2830)	4510 (3430–5920)
70 – 74 years	Male	3032 (2709–3393)	4342 (3914–4816)	6540 (5565–7679)	12,052 (9256–15,618)
	Female	1736 (1550–1944)	2494 (2246–2769)	3775 (3205–4444)	7046 (5375–9209)
75 – 79 years	Male	4697 (4201–5251)	6702 (6049–7424)	10,031 (8559–11,739)	18,184 (14,082–23,310)
	Female	2946 (2632–3297)	4220 (3804–4681)	6359 (5410–7468)	11,728 (9003–15,207)
80 – 84 years	Male	7841 (7024–8748)	11,107 (10,048–12,270)	16,422 (14,088–19,096)	28,867 (22,709–36,263)
	Female	5285 (4728–5906)	7531 (6799–8337)	11,245 (9605–13,144)	20,268 (15,743–25,882)
≥ 85 years	Male	22,118 (19,986–24,441)	30,264 (27,688–33,020)	42,258 (37,180–47,731)	64,753 (54,553–74,814)
	Female	21,912 (19,797–24,217)	29,998 (27,440–32,737)	41,923 (36,871–47,373)	64,364 (54,173–74,446)

Values are 1-year death probabilities converted from annual hazards. Age-group mortality probabilities​ from the German 2022/24 period life tables were converted to annual hazards h_x_ =  − ln(1 − nq_x_)/n. Hazards were scaled by GOLD-specific hazard ratios and converted to 1-year probabilities p = 1 − exp(− h). Equivalently $p_{v} = 1 - \left( {1 - p_{0} } \right)^{{HR_{v} }}$. h denotes the HR-scaled annual hazard. nq_x_ denotes the life-table probability of dying within an age interval of length n, h_x_ the corresponding annual hazard, and p the 1-year death probability. Reported numbers equal p × 100,000. 95% CIs were derived by applying the lower/upper 95% CI bounds of the hazard ratios to the hazard and converting back to probabilities (baseline uncertainty not modelled). For 85 + , hazards were aggregated from 85–89, 90–94, 95–99, and 100 + using person-years weights Lx ≈ n(l_x_ − 0.5d_x_); 100 + was treated as a 1-year interval (sensitivity analyses showed < 1% impact).

A network plot showing the number of studies for each comparison is shown in e-Fig. 2. Between 2 (GOLD 2 vs. 3 and GOLD 3 vs. 4) and 17 (GOLD 1 vs. normal) studies were available for comparisons. Egger and Begg-Mazumdar tests did not indicate funnel-plot asymmetry. The Thompson-Sharp test was significant in the overall comparison-adjusted funnel plot (p = 0.013), but not in the GOLD 1-comparator (p = 0.066) or normal-spirometry-comparator (p = 0.285) analyses (e-Fig. 3), providing rather mixed evidence of small-study effects. Statistical heterogeneity was substantial across most analyses (Table 3).

Net heat diagnostics (e-Fig. 4) indicated localized inconsistency signals in the core part of the network, particularly around comparisons using normal as the reference. Outside this area, contributions were small and no consistent large-scale pattern was observed. Overall, network estimates appeared stable.

The approximate relative weight per study for each GOLD grade is shown in forest plots (e-Fig. 5 and e-Fig. 6). For normal spirometry as reference, the three largest studies contributed between 30 and 65% to the total weight per GOLD grade. For GOLD 1 as reference, the corresponding range was 37%-61%. However, only studies with normal spirometry or GOLD 1 as reference were considered in this approximation (as opposed to the network meta-analysis).

### Mortality estimates

German age- and sex-specific mortality from the 2022/24 period life tables, stratified by age and sex (e-Table 5), served as the baseline for the normal-spirometry reference. Combining this baseline with pooled HRs (Table 3) yielded grade-, age-, and sex-specific 1-year mortality risks for Germany (Table 4), ranging from 0.15% (females, GOLD 1, ages 45–49) to 64.5% (males, GOLD 4, age ≥ 85). In a sensitivity analysis using the 2023/25 period life tables instead, the resulting GOLD-specific mortality probabilities changed only slightly, with a maximum absolute difference of 943 per 100,000 individuals and a maximum relative difference of 4.6%. In a second sensitivity analysis using the older 2017/19 period life table, the maximum absolute difference from the main 2022/24 estimates was 1,079 per 100,000 individuals and the maximum relative difference was 9.0% (e-Table 6). The overall interpretation was unchanged in both sensitivity analyses. To illustrate transferability, we added a worked example using Austrian 2022/24 smoothed period life-table mortality. Replacing the German baseline mortality with Austrian age- and sex-specific mortality while applying the same pooled GOLD-specific hazard ratios yielded illustrative mortality estimates calibrated to Austrian baseline mortality (e-Table 7).

## Discussion

In this network meta-analysis, we investigated the association between COPD severity (defined by GOLD grades 1–4) and all-cause mortality. Pooled hazard ratios for grades 1, 2, 3, and 4 compared to normal spirometry were 1.09, 1.57, 2.39, and 4.54, respectively. Linking the pooled HRs to German life-table mortality (Destatis) yielded age- and sex-specific annual mortality risks for each GOLD grade. While this study focused on Germany, mortality risks can be recalculated for other countries by combining the pooled HRs with local baseline mortality. However, HRs may differ across settings due to differences in case mix, smoking prevalence, and healthcare context.

We used all-cause mortality because it aligns with the model by Menn et al.^6^, is more consistently reported than cause-specific mortality, and is less prone to misclassification in the presence of multimorbidity.

While our study resulted in reasonably precise estimates of grade-specific mortality in COPD, there are several sources of heterogeneity which could not be addressed in our meta-analysis. These include more detailed smoking exposure (pack-years rather than current/former/never smoking), differences in healthcare systems, changes in treatment over time (e.g. potentially better adherence to guidelines over time), and differences in inclusion and exclusion criteria (e.g. exclusion of patients with recent exacerbation). These factors could not be examined reliably with the available study-level data. For example, Guo et al.^29^ was an influential outlier, possibly due to its younger screening population, pre-bronchodilator spirometry, extensive adjustment, and use of natural-cause rather than all-cause mortality, all of which may have attenuated hazard ratios. Consistent with this, stratification by study setting showed slightly stronger associations between GOLD grade and mortality in clinical settings. This was expected because patients from a clinical setting are expected to be sicker (more comorbidities etc.) on average (different case mix).

We included HRs adjusted at minimum for age, sex, and smoking (when comparing to normal spirometry). Adjustment sets differed across studies, but evidence suggests limited incremental impact beyond these core covariates^22,32^. Adjusting for age and sex was required to reduce confounding and support application of the pooled hazard ratios to age- and sex-specific baseline mortality. Excluding studies that adjusted for physical activity resulted in modestly higher pooled estimates, which may indicate some attenuation due to over-adjustment, although this difference may have been driven partly by the influential outlying study by Guo et al. Our framework does not estimate separate mortality risks for smokers and non-smokers. While included studies adjusted the GOLD–mortality association for smoking to reduce confounding, the baseline mortality used for calibration is not smoking-stratified. Thus, derived absolute risks should be interpreted as population-averaged. Extending the framework to smoking-stratified baseline mortality (and potentially smoking-specific hazard ratios) would be a useful direction for future work.

It should also be noted that patient composition may be heterogeneous even with identical reference groups, depending on the study setting, in- and exclusion criteria. For example, “lung-healthy” participants might comprise truly healthy individuals but also patients with diseases other than COPD. Hazard ratios would tend to be underestimated (overestimated) if the reference group is less (more) healthy than the average population.

Our study has several strengths. A key advantage of the network meta-analysis is that it allowed inclusion of HRs irrespective of the reference category. Weighted least squares (WLS) with study-clustered standard errors^47^ was used to address within-study correlation between GOLD grade-specific estimates. We focused on adjusted HRs (rather than absolute rates) because HRs account for confounding, baseline mortality varies across populations and absolute rates were reported only in a minority of studies. We conducted a systematic literature search in two data bases and were able to include articles on additional large-scale studies after obtaining all relevant information from the corresponding authors. Robustness of the results and risk of bias were assessed by sensitivity analyses and NOS assessment, respectively.

Our study has limitations. First, while we report confidence intervals for pooled hazard ratios, we did not estimate prediction intervals for setting-specific effects. A Bayesian hierarchical network meta-analysis could provide prediction intervals and more directly quantify predictive uncertainty for future applications. Second, uncertainty in the German baseline mortality was not propagated (life-table mortality treated as fixed), so uncertainty intervals for derived risks reflect only uncertainty in the hazard ratios. Third, for mortality risks in the reference group with normal spirometry, we used all-cause mortality data for the German general population. While these data inevitably include some individuals with COPD, the resulting bias is expected to be small given the comparatively low prevalence in the overall population. Lastly, the possibility of having missed a relevant article, the age of some of the included studies, and potential reporting bias need to be mentioned as potential limitations.

Throughout, we used the spirometry-based GOLD 2007 grades (1–4) to match the structure of the decision model. While later GOLD classifications (e.g., A–D, GOLD 2011/2017) incorporate symptom burden and exacerbation history^48^, the GOLD 1–4 grading showed better prediction of mortality, risk of acute exacerbations, and health-related quality of life measures. Gedebjerg et al.^24^ compared 1-year mortality across GOLD 2017 groups (A–D) and GOLD 2007 grades (1–4) in a large Danish COPD cohort. The gradient was steeper across A–D (2.3%, 6.6%, 5.2%, 11.6%) than across 1–4, likely because exacerbations are incorporated in the 2017 framework. Mortality increased monotonically across GOLD 2007 grades (3.4%, 5.3%, 8.7%, 10.9%), supporting the continued relevance of spirometry-based grading.

We excluded studies with PRISm in the reference group because PRISm is associated with higher mortality than truly lung-healthy individuals, which would bias HRs toward the null (29,34,49). While some cohorts reported PRISm and COPD in parallel^32,33^, inconsistent terminology over time (e.g., GOLD 0/X/unclassified) complicates comprehensive identification of all potentially relevant studies^49^. The only German population-based study reporting grade-specific HRs to our knowledge is LUSI^33^. Differences in inclusion criteria (e.g., heavy smoking history) may partly explain its higher mortality estimates. A recent meta-analysis by Zhao et al.^50^ also investigated mortality in patients with COPD, but differed in eligibility criteria and did not compare mortality to GOLD 1. Our meta-analysis included several studies with long follow-up and/or large case numbers which potentially did not fulfil their inclusion criteria (e.g.^13,14,18,19,30,39,41,44^).

Heterogeneity remained substantial and likely reflects differences in case mix, healthcare context, follow-up, spirometry assessment (pre/post bronchodilator), reference group definitions, and adjustment sets across cohorts. We considered formal study-level meta-regression using mean age and smoking prevalence. However, these variables were inconsistently reported and were often unavailable for the specific GOLD grade or comparison group contributing the hazard ratio. Moreover, several network contrasts were informed by only a few studies, limiting the number of comparable study-level observations. We therefore considered meta-regression insufficiently powered and potentially misleading. Instead, heterogeneity was explored through subgroup analyses by study setting and follow-up duration, leave-one-out sensitivity analyses, and inconsistency diagnostics. These analyses did not identify a single study or subgroup that explained the observed heterogeneity. Pooled estimates should therefore be interpreted with caution.

## Conclusions

This study provides a harmonized set of mortality inputs for GOLD-grade-based COPD decision models. Published cohort studies report mortality by GOLD grade using different reference groups, adjustment sets, and severity contrasts, which limits their direct use in state-transition models. By combining these estimates in a network meta-analysis, we expressed GOLD 1-4 mortality on a common scale and linked the resulting hazard ratios to German age- and sex-specific life-table mortality.

 The resulting probabilities can be used as annual mortality inputs for German Markov models with GOLD 1-4 health states. They allow mortality to vary by disease grade, age, and sex, which is important for long-term cost-effectiveness analyses where survival assumptions influence projected life-years, QALYs, and costs.

Compared with previous meta-analytic work on COPD mortality, the contribution of this study is its focus on model-ready mortality inputs rather than on the overall prognostic association alone. The same calculation framework can be applied to other countries when suitable national mortality tables are available, as illustrated with Austrian life-table data. However, absolute risks should be recalibrated to local baseline mortality and interpreted in light of local COPD case mix and healthcare context.

## Supplementary Information

Supplementary Information 1.

Supplementary Information 2.

## Data Availability

The analysis codes for R and the data (extraction sheet) to reproduce the findings can be made available upon reasonable request (i.e., research proposal) from the corresponding author.

## Abbreviations

BD: Bronchodilator
BMI: Body Mass Index
CI: Confidence interval
Destatis: German Federal Statistical Office
COPD: Chronic obstructive pulmonary disease
FEV_1_: Forced expiratory volume in 1 s
% predicted: Percentage of predicted normal
FVC: Forced vital capacity
GOLD: Global Initiative for Chronic Obstructive Lung Disease
GP: General practitioner
HR: Hazard ratio
MR: Mortality rate
NOS: Newcastle–Ottawa Scale
pop.: Population
pat.: Patient(s)
PRISMA: Preferred Reporting Items for Systematic reviews and Meta-Analyses
spiro.: Spirometry

## Code availability

Study registration: Not prospectively registered. A versioned protocol documenting the final methods has been deposited on OSF (https://osf.io/zwky4/overview?view_only=c3712d3a845740eca968ccdaefc5c5ae).
