# Supplementary material for: Estimation of mortality hazard ratios by COPD grade using a systematic review and network meta-analysis: deriving grade-, sex-, and age-specific mortality for a German COPD decision model
Source: Sci Rep. 2026 Sep 9;16:28241. doi: 10.1038/s41598-026-68641-0 (PMC13558551; doi:10.1038/s41598-026-68641-0)
Supplement: Supplementary file 2 [file 41598_2026_68641_MOESM2_ESM.docx]

**Supplementary material for the article:**

Estimation of Mortality Hazard Ratios by COPD Grade Using a Systematic Review and Network Meta-Analysis: Deriving Grade-, Sex-, and Age-Specific Mortality for a German COPD Decision Model

Tobias Niedermaier^a^ PhD, Ulrich Mansmann^a^ PhD, professor in biostatistics, and Rolf Holle^a^ PhD, professor in biostatistics.

^a^ Institute for Medical Information Processing, Biometry and Epidemiology (IBE), Faculty of

Medicine, LMU, Munich, Germany, Marchioninistr. 15, 81377 München

Short title/running head: Network meta-analysis COPD mortality by GOLD grade

Corresponding author: Tobias Niedermaier, [tobias.niedermaier@ibe.med.uni-muenchen.de](mailto:tobias.niedermaier@ibe.med.uni-muenchen.de), Marchioninistr. 15, 81377 München, Germany.

**Online Resource 1: PubMed search.**

(COPD[tiab]

OR "chronic obstructive pulmonary disease"[tiab]

OR "airflow limitation"[tiab]

OR "Pulmonary Disease, Chronic Obstructive/mortality"[Majr])

AND

(mortality[tiab] OR death[tiab] OR survival[tiab])

AND

(cohort[tiab]

OR prospective[tiab]

OR longitudinal[tiab]

OR "follow-up"[tiab]

OR "followed up"[tiab]

OR followed[tiab]

OR retrospective[tiab]

OR registry[tiab]

OR biobank[tiab]

OR screening[tiab])

AND

(GOLD[tiab]

OR "Global Initiative for Chronic Obstructive Lung Disease"[tiab]

OR stage*[tiab]

OR severit*[tiab]

OR grade*[tiab]

OR classificat*[tiab]

OR spirometr*[tiab]

OR "airflow obstruction"[tiab]

OR FEV1[tiab]

OR FVC[tiab]

OR "FEV1/FVC"[tiab]

OR PRISm[tiab]

OR ABCD[tiab])

AND

(hazard[tiab]

OR risk[tiab]

OR "hazard ratio"[tiab]

OR HR[tiab]

OR HRs[tiab]

OR "relative risk"[tiab]

OR RR[tiab]

OR RRs[tiab]

OR "odds ratio"[tiab]

OR "odds ratios"[tiab]

OR "C-statistic"[tiab]

OR "C statistic"[tiab])

The last search was conducted on February 10, 2026 and yielded 2503 results, including 33 of the 34 included articles. One article (Lundbäck 2009) was found via cross-referencing.

**Online Resource 2: e-Figure 1**: Structure of the modified Markov model

**Online Resource 3: e-Figure 2**: Network plot

The numbers in the plot correspond to the number of studies included in the respective comparison (e.g. 6 studies comparing mortality in GOLD 4 to GOLD 1). Those numbers are also represented by the thickness of the connecting lines between the health states.

**Online Resource 4: e-Figure 3**: Funnel plot of the hazard ratios for all-cause mortality.

**Online Resource 5: e-Figure 4.** Netheat plot.

Black-and-white net-heat matrix for diagnosing inconsistency in a network meta-analysis. Each cell combines a **network estimate** (row) with a **design/direct evidence component** (column). The **size of the inner square** represents the **contribution** of the column’s direct evidence to the corresponding network estimate (larger square = larger influence). The **number** is the net-heat value (change in inconsistency when that design is “detached”): **positive values** indicate that inconsistency **decreases** when the consistency assumption for that design is relaxed (suggesting a potential inconsistency “hotspot”); **negative values** indicate that inconsistency **increases** (the design tends to support overall consistency). Cells with **large squares and large positive values** are most informative, as they point to influential evidence components that may be driving inconsistency.

Large square + large positive number (high contribution, strong “hotspot” signal):
The direct evidence in that column strongly influences the row’s network estimate, and detaching that design would substantially reduce inconsistency → high-priority candidate for driving inconsistency.

Large square + number near 0 (high contribution, little inconsistency signal):
The design has high influence on the network estimate, but detaching it changes inconsistency hardly at all → important evidence for the estimate, but not a likely inconsistency driver.

**Small square + large positive number** (low contribution, strong local signal):
Detaching the design would **reduce inconsistency**, but the design contributes **little** to the network estimate → potentially a **local** inconsistency issue, but usually lower priority than “large + large.”

**Small square + number near 0** (low contribution, no inconsistency signal):
The design has **minimal influence** on the network estimate, and detaching it changes inconsistency **negligibly** → typically **low relevance** for both effect estimation and inconsistency.

**Online Resource 6: e-Figure 5.** Forest plot of hazard ratios (95% confidence intervals) compared to normal spirometry.

**Online Resource 7: e-Figure 6.** Forest plot of hazard ratios (95% confidence intervals) compared to GOLD 1.

e-Table 1: Risk of bias assessment (Newcastle-Ottawa Scale)

| Study (first author, year, ref.) | Selection | | | | Comparability | Outcome | | |
| --- | --- | --- | --- | --- | --- | --- | --- | --- |
|  | **Representativeness of the Exposed Cohort** | **Selection of the Non-Exposed Cohort** | **Ascertainment of Exposure [✓ for spirometry with bronchodilator]** | **Demonstration That Outcome of Interest Was Not Present at start of Study** | **Comparability of Cohorts on the Basis of the Design or Analysis^1^** | **Assessment of Outcome** | **Was Follow-Up Long Enough for Outcomes to Occur** | **Adequacy of Follow Up of Cohorts** |
| **Mannino 2003 [1]** | ✓ (NHANES I) | ✓ (NHANES I) | (✓) (spiro without bronchodilator) | ✓ (by definition [mortality]) | ✓✓ (age, sex, race, smo., edu., BMI) | (✓) [death certificates] | ✓ (17-22 years) | ✓ (96%) |
| **Ekberg-Aronsson 2005 [2]** | ✓ (pop.-wide screening) | ✓ (pop.-wide screening) | (✓) (spirometry, BD unclear) | ✓ (by definition [mortality]) | ✓ (age, inhal., smoking-adj.) | ✓ (mortality registers) | ✓ (11-29 years) | ✓ (record linkage) |
| **Mannino 2006 [3]** | ✓ (ARIC) | ✓ (ARIC) | (✓) (spiro without bronchodilator) | ✓ (by definition [mortality]) | ✓✓ (age, sex, smo., BMI, race, edu.) | (✓) [death certificates] | ✓ (up to 11 years) | ✓ (avail. for 97.9% of the cohort) |
| **Stavem 2006 [4]** | ̶ (apparently healthy **men**) | ✓ (same community) | (✓) (spiro without bronchodilator) | ✓ (by definition [mortality]) | ✓✓ (age, smo, FEV1, chol., BP, fitness, BMI) | ✓ (mortality registers) | ✓ (average 26 years) | ✓ (range 25-27 years) |
| **Lundbäck 2009 [5]** | ✓ (questionnaire survey in northern Sweden) | ✓ (questionnaire survey in northern Sweden) | (✓) (spiro without bronchodilator) | ✓ (by definition [mortality]) | ✓✓ (age, sex, smoking, heart disease) | ✓ (population register) | ✓ (20 years) | ✓ (median 18 years) |
| **Alfageme 2010 [6]** | ̶ (outpatients) | ✓ (same community) | (✓) (spirometry, BD unclear) | ✓ (by definition [mortality]) | ✓✓ (age, smo., GOLD etc.) | ✓ (pat. records, relatives, caregivers, doctors etc.) | ̶ (3 years) | ✓ (4 of 600 pat. dropped out) |
| **Mannino 2011 [7]** | (✓) (Lung Health Study, “healthy” smo.) | ✓(Lung Health Study) | ✓ (pre- + post-BD spirometry) | ✓ (by definition [mortality]) | ✓✓ (age, sex, smo,BMI,educ.,…) | ✓ [biannual phone contacts] | ✓ (10 years, up to 14.5 years) | - [~75%] |
| **Mannino 2012 [8]** | ✓ (NHANES III) | ✓ (NHANES III) | (✓) (spiro without bronchodilator) | ✓ (by definition [mortality]) | ✓✓ (age,sex,race, BMI,smo,diab,CVD.. | ✓ (mortality registers) | ✓ (12-18 years) | ✓ (only 4 without mortality data) |
| **Johannessen 2013 [9]** | (✓)(hospital patients + general pop.) | ̶ (hospital patients + general pop.) | ✓ (spirometry) | ✓ (by definition [mortality]) | ✓✓ (age, sex, smo,, BMI, comorb.) | ✓ (mortality registers) | ✓ (8.5 years) | (✓) [~80%] |
| **Leivseth 2013 [10]** | ✓ (HUNT2) | ✓ (HUNT2) | ✓ (spirometry) | ✓ (by definition [mortality]) | ✓✓ (age,smo, edu,BMI,PA,CVD,…) | ✓ (mortality registers) | ✓ (up to 17 years) | ✓ (median 14.6 years) |
| **Mattila 2015 [11]** | ✓ (nat. repr. sample) | ✓ (nat. repr. sample) | (✓) (spiro. without bronchodil.) | ✓ (by definition [mortality]) | ✓ (age, sex) | ✓ (indiv. ident. number) | ✓ (28-30 years) | ✓ (record linkage) |
| **Brusse-Keizer 2017 [12]** | - (patients who smoke(d)) | ✓ (same community) | (✓) (spirometry, BD unclear) | ✓ (by definition [mortality]) | ✓ (age, sex, BMI, comorb.) | ✓ (public registries) | ̶ (3 years) | ✓ (registers, 665/795 with complete data) |

**e-Table 1, continued.**

| Study (first author, year, ref.) | Selection | | | | Comparability | Outcome | | |
| --- | --- | --- | --- | --- | --- | --- | --- | --- |
|  | **Representativeness of the Exposed Cohort** | **Selection of the Non-Exposed Cohort** | **Ascertainment of Exposure [✓ for spirometry with bronchodilator]** | **Demonstration That Outcome of Interest Was Not Present at Start of Study** | **Comparability of Cohorts on the Basis of the Design or Analysis^1^** | **Assessment of Outcome** | **Was Follow-Up Long Enough for Outcomes to Occur** | **Adequacy of Follow Up of Cohorts** |
| **Gedebjerg 2018** [13] | ̶ (outpatients) | ✓ (same community) | (✓) (spirometry, BD unclear) | ✓ (by definition [mortality]) | ✓✓ (age, sex, marital status, comorb., BMI, smo., CVD medication | ✓ (mortality registers) | ̶ (3 years) | ✓ (record linkage) |
| **Perez-Padilla 2018** [14] | ✓ (pop.-based surveys) | ✓ (pop.-based surveys) | ✓ (spirometry) | ✓ (by definition [mortality]) | ✓✓ (age,sex,BMI, educ.,comorb.,smo.) | ? (not specified) | ✓ (9 years) | (✓) (~80%) |
| **Wijnant 2020** [15] | ✓ (pop.-based cohort) | ✓ (pop.-based cohort) | (✓) (spiro. without bronchodilator) | ✓ (by definition [mortality]) | ✓✓ (age, sex, smo, BMI) | ✓(GPs+municipal records) | ✓ (9.8 years) | ✓ (record-based follow-up) |
| **Brat 2021** [16] | (✓) (unselected pop. of COPD pat.) | ✓ (same community) | ✓ (spirometry, BD unclear) | ✓ (by definition [mortality]) | ✓ (age, sex) | ✓(?) | ✓ (5 years) | (✓) (720/784 complete data) |
| **Damkjaer 2021** [17] | (✓) (single-center cohort) | ✓ (same pat. with COPD diag) | (✓) (prescription registry) | ✓ (by definition [mortality]) | ✓✓ (age, sex, smoking) | ✓ (civil registry) | ✓ (3.5 years) | ✓ (civil registry) |
| **Guo 2021** [18] | (✓) (selected screening cohort) | ✓ (same community) | (✓) (spirometry without bronchodilator) | ✓ (by definition [mortality]) | ✓✓ (age, sex, educ., BMI, smo., alc., PA, diet, PM_2.5_ etc.) | ✓ (national death registry) | ✓ (median 16 years) | ✓ (record linkage) |
| **Hasenstab 2021** [19] | (✓) (COPDGene) | ✓ (same community) | ✓ (spirometry) | ✓ (by definition [mortality]) | ✓✓ (age, sex, race, smoking) | ✓ (mortality follow-up every 6 months) | ✓ (10 years) | ? (not reported) |
| **He 2021** [20] | ✓ (pop.-based cohort | ✓ (same community) | ✓ (spirometry without bronchodilator) | ✓ (by definition [mortality]) | ✓✓ (age, sex, marital status, educ., BMI, baseline CVD+cancer, smo., drinking, PA | (✓) (end of life interviews) | ✓ (7.7 years) | ? (not reported) |
| **Washio 2022** [21] | ✓ (pop.-based screening) | ✓ (pop.-based screening) | (✓) (spiro without bronchodilator) | ✓ (by definition [mortality]) | ✓✓ (age, sex, smo, BMI, diab., CVD, cancer etc.) | ✓ (death certificates) | ✓ (median 5.3 years) | ✓ (no loss-to-follow-up) |
| **Kaaks 2022** [22] | (✓) (patients who smoke(d)) | ✓ (same community) | (✓) (spiro without bronchodilator) | ✓ (by definition [mortality]) | ✓✓ (age, sex, smo) | ✓ (record linkage, mort. reg.) | ✓ (median 12.1 years) | ✓ (record linkage) |
| **Cheng 2023** [23] | - (outpatients) | ✓ (same) | ✓ (spirometry) | ✓ (by definition) | ✓✓(age,sex,smo,BMI) | ✓ (indiv. follow-up) | (✓) (3-6 years) | ? (not reported) |
| **Cadham 2024** [24] | ✓ (NHANES) | ✓ (same) | (✓) (spiro without bronchodilator) | ✓ (by definition [mortality]) | ✓✓(age,sex,smo,BMI, race) | ✓ (record linkage) | ✓ (mean 9.4 years) | ✓ (record linkage) |

**e-Table 1, continued.**

| Study (first author, year, ref.) | Selection | | | | Comparability | Outcome | | |
| --- | --- | --- | --- | --- | --- | --- | --- | --- |
|  | **Representativeness of the Exposed Cohort** | **Selection of the Non-Exposed Cohort** | **Ascertainment of Exposure [✓ for spirometry with bronchodilator]** | **Demonstration That Outcome of Interest Was Not Present at Start of Study** | **Comparability of Cohorts on the Basis of the Design or Analysis^1^** | **Assessment of Outcome** | **Was Follow-Up Long Enough for Outcomes to Occur** | **Adequacy of Follow Up of Cohorts** |
| **Whittaker 2024** [25] | ✓ (healthcare record🡪pop.wide) | ✓ (same) | (✓) (clinical diagnosis of COPD) | ✓ (by definition [mortality]) | ✓✓ (age, sex, smo, BMI, depr., diab., etc.) | ✓ (national death registry) | ✓ (mean 5.5 years) | (✓) (record linkage, but pot. censoring) |
| **Egerod 2025** [26] | - (cases referred with severe COPD) | ✓ (same) | ✓ (spirometry) | ✓ (by definition [mortality]) | ✓✓ (age, sex, smoking) | ✓ (record linkage, see cohort profile) | (✓) (3 years) | ✓ (record linkage) |
| **Hyung 2025** [27] | - (multicenter, clinic-based) | ✓ (same) | ✓ (spirometry) | ✓ (by definition [mortality]) | ✓✓ (age, sex, smo, BMI, asthma, CVD, baseline treatments) | ✓ (national death registry) | ✓ (median 5 years) | ✓ (record linkage) |
| **Ke 2025** [28] | (✓) (~30% response, large prosp. study) | (✓) (same) | ✓ (spirometry) | ✓ (by definition [mortality]) | ✓✓ (age, sex, BMI, smoking, asthma, CVD) | ✓ (national death registry) | ✓ (16 years) | ✓ (record linkage) |
| **Lin 2025** [29] | ✓ (Insurance claim database) | ✓ (same) | ✓ (spirometry) | ✓ (by definition [mortality]) | ✓✓ (age, sex, body height, weight, race) | ✓ (Insurance claim data) | ✓ (4 years) | ✓ (record linkage) |
| **Ma 2025** [30] | ✓ (UK Biobank study) | ✓ (same) | (✓) (spiro without bronchodilator) | ✓ (by definition [mortality]) | ✓✓ (age, sex, BMI, smoking, educ., …) | ✓ (UKBB Cause of Death registry) | ✓ (14-22 years) | ✓ (record linkage) |
| **Niedermaier 2025** [31] | - (multicenter, clinic-based) | ✓ (same) | ✓ (spirometry) | ✓ (by definition [mortality]) | ✓✓ (age, sex, [smoking]^2^) | ✓ (indiv. follow-up) | ✓ (6 years) | (✓) (late follow-up less reliable) |
| **Ogata 2025** [32] | - (multicenter, hospital-based) | ✓ (same) | (✓) (spiro without bronchodilator) | ✓ (by definition [mortality]) | ✓✓ (age, sex, BMI, smo., ICS, LABA/ LAMA, asthma, CRP) | ✓ (indiv. follow-up for 5 years) | ✓ (5 years) | ? (not specified) |
| **Wallström 2025** [33] | (✓) (nationwide cohort study)^3^ | ✓ (same) | ✓ (spirometry) | ✓ (by definition [mortality]) | ✓✓ (age, sex, smoking, BMI, CVD) | ✓ (national death registry) | (✓) (median 3.74 years) | ✓ (record linkage) |
| **Casanova 2026** [34] | - (multicenter, clinic-based) | ✓ (same) | ✓ (spirometry) | ✓ (by definition [mortality]) | ✓✓ (age, sex, smo, BMI) | ✓ (family and electronic medical records) | ✓ (10 years) | ✓ (family + medical records) |

^1^One point for at least age and smoking adjustment, plus one extra point if adjusted for at least one other factor. ^2^Supplementary analysis. ^3^Many exclusions due to missing spirometry. Abbreviations: alc., alcohol; BMI, body-mass index; chol., cholesterol; comorb., comorbidities; COPD, chronic obstructive pulmonary disease; CVD, cardiovascular disease; depr., depression; diab., diabetes; educ., education; GP, general practitioner; indiv., individual; inhal., tobacco smoke inhalation habits (yes/no); nat. rep., nationally representative; PA, physical activity; pat., patients; pop., population; smo., smoking.

**Summary scores: (3-4 was counted as 3.5 points etc., (✓) was counted as 0.5 points, ✓ was counted as 1 point.)**

|  |  | Summary score by domain | | |
| --- | --- | --- | --- | --- |
| Study (first author, year, ref.) | **Summary score** | **Selection** | **Comparability** | **Outcome** |
| **Mannino 2003** [1] | 8 | 3-4 | 2 | 2-3 |
| **Ekberg-Aronsson 2005** [2] | 7-8 | 3-4 | 1 | 3 |
| **Mannino 2006** [3] | 8 | 3-4 | 2 | 2-3 |
| **Stavem 2006** [4] | 7-8 | 2-3 | 2 | 3 |
| **Lundbäck 2009** [5] | 8-9 | 3-4 | 2 | 3 |
| **Alfageme 2010** [6] | 6-7 | 2-3 | 2 | 2 |
| **Mannino 2011** [7] | 7-8 | 3-4 | 2 | 2 |
| **Mannino 2012** [8] | 8-9 | 3-4 | 2 | 3 |
| **Johannessen 2013** [9] | 7 | 2-3 | 2 | 2-3 |
| **Leivseth 2013** [10] | 9 | 4 | 2 | 3 |
| **Mattila 2015** [11] | 7-8 | 3-4 | 1 | 3 |
| **Brusse-Keizer 2017** [12] | 5-6 | 2-3 | 1 | 2 |
| **Gedebjerg 2018** [13] | 6-7 | 2-3 | 2 | 2 |
| **Perez-Padilla 2018** [14] | 7-8 | 4 | 2 | 1-2 |
| **Wijnant 2020** [15] | 8-9 | 3-4 | 2 | 3 |
| **Brat 2021** [16] | 7 | 3-4 | 1 | 2-3 |
| **Damkjaer 2021** [17] | 8 | 3 | 2 | 3 |
| **Guo 2021** [18] | 8 | 3 | 2 | 3 |
| **Hasenstab 2021** [19] | 7-8 | 3-4 | 2 | 2 |
| **He 2021** [20] | 7-8 | 4 | 2 | 1-2 |
| **Washio 2022** [21] | 8-9 | 3-4 | 2 | 3 |
| **Kaaks 2022** [22] | 8 | 3 | 2 | 3 |
| **Cheng 2023** [23] | 6-7 | 3 | 2 | 1-2 |
| **Cadham 2024** [24] | 8-9 | 3-4 | 2 | 3 |
| **Whittaker 2024** [25] | 8 | 3-4 | 2 | 2-3 |
| **Egerod 2025** [26] | 7-8 | 3 | 2 | 2-3 |
| **Hyung 2025** [27] | 8 | 3 | 2 | 3 |
| **Ke 2025** [28] | 8 | 3 | 2 | 3 |
| **Lin 2025** [29] | 9 | 4 | 2 | 3 |
| **Ma 2025** [30] | 8-9 | 3-4 | 2 | 3 |
| **Niedermaier 2025** [31] | 7-8 | 3 | 2 | 2-3 |
| **Ogata 2025** [32] | 6-7 | 2-3 | 2 | 2 |
| **Wallström 2025** [33] | 8 | 3-4 | 2 | 2-3 |
| **Casanova 2026** [34] | 8 | 3 | 2 | 3 |

| **e-Table 2**. Overview of seemingly relevant studies and reason for exclusion. | | |
| --- | --- | --- |
| First author, year, ref. | **HRs for mortal­ity by COPD grade reported where?** | **Main reason(s) for exclusion** |
| **Alupo 2021** [35] | Table 2 | Patients with chest symptoms undergoing clinical evaluation in a hospital. |
| **Athlin 2021** [36] | Table 3 | Not smoking adjusted, potentially critically ill patients. |
| **Backman 2024** [37] | Table 2 | Not smoking adjusted. |
| **Bavishi 2022** [38] | Table S6 | Tiffeneau index partly >0.70, not smoking adjusted; potentially critically ill patients. |
| **Bertels 2024** [39] | Text | Not smoking adjusted. |
| **Bhatt 2023** [40] | Table 1 | Not smoking adjusted. |
| **Bhatta 2020** [41] | Table 2 | Follow-up >20 years, GOLD grade might have progressed, redundant to Leivseth 2013. |
| **Borvik 2016** [42] | Table 4 | Selection towards ill patients (thromboembolism). |
| **Borvik 2020** [43] | Table 3 | Selection towards ill patients (thromboembolism). |
| **Chan 2016** [44] | Text | Hospitalized patients. |
| **Chen 2015** [45] | Table 3 | Reported odds ratios, not hazard ratios. |
| **Dusser 2016** [46] | Table S5 | Unadjusted (crude) estimates. |
| **Garcia-Aymerich 2011** [47] | Table 3 | Patients hospitalized after exacerbation. |
| **Holmgaard 2013a** [48] | Table 2 | Not adjusted for sex+hospitalized patients. |
| **Holmgaard 2013b** [49] | Table 4 | Not adjusted for sex+hospitalized patients. |
| **Hwang 2020** [50] | Table 5 | FEV1 categories not suitable (≥ vs.<50 % predicted). |
| **Lee 2014** [51] | Table 2 | Redundant to Mannino 2012. |
| **Nishimura 2024** [52] | Text | Unadjusted (crude) estimates. |
| **Pedone 2018** [53] | Table 3 | Apparently unadjusted (crude) estimates. |
| **Shavelle 2009** [54] | Table 3 | No CIs reported, redundant to Mannino 2012. |
| **Song 2022** [55] | Table 5 | Reports odds ratios instead of hazard ratios, and only in patients with GOLD B or D. |
| **Soriano 2013** [56] | Figure 3A | Not adjusted for age, sex, smoking, patient condition unclear. |
| **Soriano 2015** [57] | Table 2 | Estimates apparently not adjusted. |
| **Valvi 2012** [58] | Figure 1 | HRs cannot be extracted unambiguously, redundant to Mannino 2006. |
| **Yong 2020** [59] | Table 3 | HR reported only per GOLD grade increase |
| **Zou 2025** [60] | Table 2 | Redundant to Mannino 2012, very long follow-up (25.7 years) affects GOLD grade over time. |
| **Abbreviations: CIs, confidence intervals; HRs, hazard ratios; COPD, chronic obstructive pulmonary disease; FEV1, forced expiratory volume in 1 second.** e-Table 2 presents apparently pertinent articles that were excluded after careful full text review. | | |

**e-Table 3.** Pooled hazard ratios (95% confidence intervals) for mortality and I² when leaving out one study at a time (reference: normal spirometry)

| Study/estimate left out (first author, year, ref.): | GOLD 1 | GOLD 2 | GOLD 3 | GOLD 4 | I squared |
| --- | --- | --- | --- | --- | --- |
|  | Hazard ratio | Hazard ratio | Hazard ratio | Hazard ratio | - |
| **Mannino** **2003** [1] | 1.08 (0.96-1.22) | 1.57 (1.40-1.75) | 2.39 (2.01-2.83) | 4.53 (3.42-6.01) | 90.1 |
| **Ekberg-Aronsson** **2005** [2] | 1.08 (0.96-1.23) | 1.57 (1.40-1.77) | 2.36 (1.96-2.84) | 4.55 (3.36-6.16) | 90.3 |
| **Mannino** **2006** [3] | 1.07 (0.96-1.20) | 1.53 (1.39-1.68) | 2.36 (2.01-2.78) | 4.48 (3.39-5.92) | 89.6 |
| **Stavem** **2006** [4] | 1.08 (0.96-1.22) | 1.56 (1.40-1.74) | 2.40 (2.02-2.85) | 4.53 (3.42-6.00) | 90.2 |
| **Lundbäck** **2009** [5] | 1.09 (0.97-1.22) | 1.57 (1.41-1.74) | 2.39 (2.02-2.83) | 4.51 (3.41-5.98) | 90.2 |
| **Alfageme** **2010** [6] | 1.09 (0.97-1.22) | 1.56 (1.41-1.74) | 2.39 (2.02-2.83) | 4.58 (3.43-6.11) | 90.0 |
| **Mannino** **2011** [7] | 1.09 (0.97-1.23) | 1.57 (1.41-1.76) | 2.39 (2.02-2.84) | 4.55 (3.43-6.03) | 90.1 |
| **Mannino** **2012** [8] | 1.08 (0.96-1.22) | 1.56 (1.39-1.74) | 2.38 (2.01-2.82) | 4.53 (3.41-6.00) | 90.0 |
| **Johannessen** **2013** [9] | 1.09 (0.97-1.22) | 1.59 (1.43-1.75) | 2.38 (2.00-2.82) | 4.48 (3.35-5.98) | 90.1 |
| **Leivseth 2013** [10] | 1.10 (0.98-1.23) | 1.56 (1.41-1.74) | 2.36 (2.00-2.80) | 4.53 (3.41-6.03) | 90.2 |
| **Mattila 2015** [11] | 1.10 (0.97-1.25) | 1.59 (1.41-1.79) | 2.47 (2.06-2.95) | 4.63 (3.43-6.24) | 90.2 |
| **Brusse-Keizer 2017** [12] | 1.10 (0.98-1.23) | 1.57 (1.41-1.74) | 2.37 (2.01-2.80) | 4.52 (3.41-5.99) | 90.2 |
| **Gedebjerg** **2018** [13] | 1.09 (0.97-1.23) | 1.57 (1.41-1.75) | 2.38 (2.00-2.82) | 4.52 (3.36-6.08) | 90.2 |
| **Perez-Padilla 2018** [14] | 1.08 (0.96-1.21) | 1.57 (1.41-1.74) | 2.38 (2.02-2.81) | 4.52 (3.42-5.98) | 90.0 |
| **Wijnant 2020** [15] | 1.09 (0.97-1.23) | 1.57 (1.41-1.75) | 2.40 (2.02-2.83) | 4.55 (3.43-6.02) | 90.0 |
| **Brat 2021** [16] | 1.09 (0.97-1.22) | 1.57 (1.42-1.74) | 2.39 (2.01-2.83) | 4.55 (3.40-6.09) | 90.1 |
| **Damkjaer 2021** [17] | 1.09 (0.97-1.22) | 1.57 (1.41-1.75) | 2.39 (2.02-2.83) | 4.54 (3.43-6.01) | 90.3 |
| **Guo 2021** [18] | 1.14 (1.03-1.25) | 1.62 (1.47-1.78) | 2.54 (2.21-2.91) | 5.01 (4.11-6.09) | 83.8 |
| **Hasenstab 2021** [19] | 1.08 (0.96-1.22) | 1.56 (1.39-1.76) | 2.35 (1.96-2.82) | 4.35 (3.24-5.83) | 89.7 |
| **He 2021** [20] | 1.08 (0.96-1.21) | 1.56 (1.41-1.74) | 2.38 (2.01-2.81) | 4.52 (3.41-5.97) | 89.9 |
| **Washio 2022** [21] | 1.08 (0.97-1.22) | 1.56 (1.40-1.74) | 2.39 (2.02-2.82) | 4.53 (3.43-5.99) | 90.1 |
| **Kaaks 2022** [22] | 1.09 (0.97-1.22) | 1.57 (1.41-1.75) | 2.39 (2.02-2.83) | 4.54 (3.44-6.01) | 90.0 |
| **Cheng 2023** [23] | 1.08 (0.96-1.21) | 1.57 (1.41-1.75) | 2.41 (2.03-2.85) | 4.58 (3.45-6.09) | 90.2 |
| **Cadham 2024** [24] | 1.08 (0.96-1.22) | 1.55 (1.39-1.72) | 2.38 (2.01-2.81) | 4.51 (3.41-5.97) | 90.0 |
| **Whittaker 2024** [25] | 1.09 (0.97-1.22) | 1.57 (1.41-1.75) | 2.39 (2.01-2.84) | 4.61 (3.46-6.16) | 89.0 |
| **Egerod 2025** [26] | 1.09 (0.97-1.22) | 1.57 (1.41-1.75) | 2.42 (2.05-2.85) | 4.49 (3.38-5.95) | 90.0 |
| **Hyung 2025** [27] | 1.09 (0.97-1.22) | 1.57 (1.41-1.75) | 2.39 (2.01-2.83) | 4.54 (3.42-6.02) | 90.3 |
| **Ke 2025** [28] | 1.10 (0.97-1.25) | 1.60 (1.42-1.80) | 2.43 (2.00-2.95) | 4.69 (3.44-6.38) | 89.8 |
| **Lin 2025** [29] | 1.07 (0.96-1.19) | 1.58 (1.42-1.76) | 2.42 (2.03-2.89) | 4.68 (3.46-6.33) | 88.2 |
| **Ma 2025** [30] | 1.09 (0.96-1.23) | 1.57 (1.40-1.76) | 2.39 (2.02-2.83) | 4.55 (3.43-6.02) | 89.3 |
| **Niedermaier 2025** [31] | 1.10 (0.99-1.23) | 1.57 (1.41-1.74) | 2.37 (2.00-2.80) | 4.44 (3.36-5.87) | 90.0 |
| **Ogata 2025** [32] | 1.09 (0.97-1.22) | 1.58 (1.42-1.75) | 2.41 (2.04-2.84) | 4.45 (3.37-5.88) | 90.2 |
| **Wallström 2025** [33] | 1.10 (0.98-1.23) | 1.57 (1.41-1.75) | 2.37 (1.99-2.81) | 4.46 (3.32-5.99) | 89.7 |
| **Casanova 2026** [34] | 1.09 (0.97-1.22) | 1.57 (1.41-1.75) | 2.38 (2.02-2.82) | 4.55 (3.43-6.02) | 90.3 |

| **e-Table 4**. Results of network meta-analysis / sensitivity analyses | | | | |
| --- | --- | --- | --- | --- |
| Grade | **Pooled hazard ratio (95% CI), reference normal** | | | |
|  | **All studies^1^** | **Population studies^1^** | **Clinical setting^2^** | **Population studies^2^** |
| ***Including Bhatta 2020 instead of Leivseth 2013 for the HUNT study, and including Zou 2025 instead of Mannino 2012 for NHANES III, GOLD 1*** | | | | |
| **Normal** | Ref. | Ref. | NA | 0.87 (0.80-0.94) |
| **GOLD 1** | 1.10 (0.97-1.23) | 1.15 (1.06-1.25) | Ref. | Ref. |
| **GOLD 2** | 1.56 (1.40-1.74) | 1.62 (1.46-1.80) | 1.40 (1.20-1.63) | 1.41 (1.28-1.56) |
| **GOLD 3** | 2.36 (1.99-2.80) | 2.19 (1.64-2.92) | 2.28 (1.85-2.81) | 1.91 (1.41-2.57) |
| **GOLD 4** | 4.53 (3.40-6.04) | 3.93 (1.69-9.16) | 4.46 (3.29-6.04) | 3.42 (1.63-7.21) |
| **Heterogeneity (I²)** | 90.3% | 91.5% | 85.6% | 91.5% |
|  | | | | |
| ***Excluding studies that adjusted for physical activity at baseline (Stavem, He, Guo, Ke)*** | | | | |
| **Normal** | Ref. | Ref. | NA | 0.89 (0.82-0.97) |
| **GOLD 1** | 1.15 (1.04-1.27) | 1.12 (1.04-1.21) | Ref. | Ref. |
| **GOLD 2** | 1.65 (1.50-1.83) | 1.69 (1.50-1.89) | 1.40 (1.20-1.63) | 1.50 (1.36-1.66) |
| **GOLD 3** | 2.62 (2.26-3.04) | 2.58 (1.69-3.94) | 2.28 (1.85-2.81) | 2.30 (1.54-3.44) |
| **GOLD 4** | 5.21 (4.28-6.34) | 5.64 (1.60-19.90) | 4.46 (3.29-6.04) | 5.03 (1.97-12.87) |
| **Heterogeneity (I²)** | 80.2% | 65.6% | 85.6% | 65.6% |
|  | | | | |
| ***Excluding studies that adjusted for medication (Hyung, Ogata)*** | | | | |
| **Normal** | Ref. | Ref. | NA | 0.90 (0.82-0.99) |
| **GOLD 1** | 1.09 (0.97-1.22) | 1.11 (1.01-1.22) | Ref. | Ref. |
| **GOLD 2** | 1.58 (1.42-1.75) | 1.63 (1.48-1.81) | 1.40 (1.18-1.67) | 1.47 (1.32-1.65) |
| **GOLD 3** | 2.40 (2.03-2.84) | 2.30 (1.76-2.99) | 2.30 (1.82-2.90) | 2.07 (1.55-2.75) |
| **GOLD 4** | 4.44 (3.35-5.89) | 4.06 (2.01-8.19) | 4.33 (3.15-5.95) | 3.65 (1.96-6.81) |
| **Heterogeneity (I²)** | 90.5% | 91.1% | 87.6% | 91.1% |
|  | | | | |
| **Abbreviations: CI = confidence interval; GOLD = Global Initiative for Chronic Obstructive Lung Disease; normal = normal spirometry. ^1^Reference: normal spirometry. ^2^Reference: GOLD 1. *^3^Dichotomized at the median of reported follow-up durations among studies reporting mean or median follow-up.*** | | | | |
| **e-Table 4 presents the pooled hazard ratios (95% CIs) with reference group normal by GOLD grade, obtained from network meta-analysis, conducted using a random effects model. All included studies were considered in the analyses regardless of their reference group.** | | | | |

| **e-Table 5.** German 2022/24 period life-table baseline mortality by sex and age group | | | | | | | | | |
| --- | --- | --- | --- | --- | --- | --- | --- | --- | --- |
|  | **45- <50 years** | **50- <55 years** | **55- <60 years** | **60- <65 years** | **65- <70 years** | **70- <75 years** | **75- <80 years** | **80- <85 years** | **85+ years** |
| **Male** | 240 | 401 | 674 | 1158 | 1851 | 2788 | 4322 | 7224 | 20512 |
| **Female** | 134 | 222 | 365 | 622 | 1011 | 1596 | 2709 | 4864 | 20320 |

| e-Table 6: 1-year mortality risks (95% CI) per 100,000 individuals in Germany, stratified by sex, age group, and GOLD grade, using the German 2017/19 period life table as baseline mortality. | | | | | | |  |
| --- | --- | --- | --- | --- | --- | --- | --- |
|  | **Age group** | **Sex** | **GOLD 1** | **GOLD 2** | **GOLD 3** | **GOLD 4** |  |
|  | 45 – 49 years | Male | 259 (231-291) | 374 (336-416) | 569 (482-672) | 1078 (816-1423) |  |
|  |  | Female | 149 (133-167) | 215 (193-239) | 327 (277-386) | 620 (469-819) |  |
|  | 50 – 54 years | Male | 447 (399-501) | 644 (580-716) | 980 (830-1157) | 1852 (1404-2442) |  |
|  |  | Female | 253 (225-283) | 364 (327-405) | 554 (469-654) | 1050 (795-1386) |  |
|  | 55 – 59 years | Male | 781 (697-875) | 1124 (1011-1249) | 1707 (1447-2014) | 3217 (2443-4232) |  |
|  |  | Female | 421 (375-472) | 606 (545-674) | 922 (781-1089) | 1744 (1322-2299) |  |
|  | 60- 64 years | Male | 1286 (1148-1440) | 1849 (1664-2053) | 2803 (2378-3303) | 5255 (4000-6889) |  |
|  |  | Female | 678 (605-760) | 977 (879-1086) | 1485 (1258-1751) | 2800 (2125-3686) |  |
|  | 65 – 69 years | Male | 1959 (1749-2193) | 2812 (2532-3121) | 4252 (3612-5004) | 7920 (6049-10337) |  |
|  |  | Female | 1042 (930-1167) | 1499 (1349-1665) | 2274 (1929-2681) | 4275 (3250-5613) |  |
|  | 70 – 74 years | Male | 2894 (2585-3238) | 4146 (3736-4599) | 6248 (5315-7338) | 11529 (8848-14954) |  |
|  |  | Female | 1655 (1477-1853) | 2377 (2140-2640) | 3599 (3055-4238) | 6723 (5127-8793) |  |
|  | 75 – 79 years | Male | 4471 (3998-4999) | 6383 (5760-7072) | 9562 (8156-11195) | 17373 (13439-22302) |  |
|  |  | Female | 2681 (2395-3001) | 3843 (3463-4264) | 5796 (4929-6811) | 10719 (8217-13922) |  |
|  | 80 – 84 years | Male | 7802 (6989-8704) | 11053 (9998-12211) | 16344 (14021-19008) | 28741 (22606-36114) |  |
|  |  | Female | 5334 (4772-5961) | 7600 (6862-8414) | 11347 (9693-13262) | 20442 (15881-26095) |  |
|  | ≥ 85 years | Male | 22455 (20294-24807) | 30698 (28093-33484) | 42806 (37684-48316) | 65385 (55170-75409) |  |
|  |  | Female | 22486 (20323-24841) | 30738 (28131-33527) | 42856 (37731-48370) | 65443 (55226-75464) |  |
| Values are 1-year death probabilities per 100,000 individuals. Age-group mortality probabilities from the German 2017/19 period life table were converted to annual hazards h_x_=−ln(1−_n_q_x_)/n. Hazards were scaled by the GOLD-specific hazard ratios and converted back to 1-year mortality probabilities. For ages ≥85 years, the same aggregation procedure as in the main analysis was used. Reported numbers equal p×100,000. 95% CIs were derived by applying the lower and upper 95% CI bounds of the hazard ratios; uncertainty in baseline mortality was not modelled. Source: German Federal Statistical Office (Destatis), GENESIS-Online table 12621-0001, period 2017/19. | | | | | | | |

| e-Table 7: Worked example for transferability: 1-year mortality risks (95% CI) per 100,000 individuals in Austria, stratified by sex, age group, and GOLD grade, using Austrian 2022/24 smoothed period life-table mortality. | | | | | | |  |
| --- | --- | --- | --- | --- | --- | --- | --- |
|  | **Age group** | **Sex** | **GOLD 1** | **GOLD 2** | **GOLD 3** | **GOLD 4** |  |
|  | 45 – 49 years | Male | 224 (200-251) | 322 (290-358) | 491 (416-580) | 930 (704-1228) |  |
|  |  | Female | 127 (113-142) | 183 (164-203) | 278 (236-329) | 528 (399-697) |  |
|  | 50 – 54 years | Male | 368 (328-412) | 530 (476-589) | 806 (682-951) | 1524 (1155-2011) |  |
|  |  | Female | 194 (173-218) | 280 (252-311) | 426 (361-503) | 807 (611-1066) |  |
|  | 55 – 59 years | Male | 619 (552-693) | 891 (801-990) | 1354 (1147-1597) | 2555 (1938-3365) |  |
|  |  | Female | 319 (284-357) | 459 (413-511) | 699 (592-825) | 1323 (1002-1746) |  |
|  | 60- 64 years | Male | 1072 (957-1201) | 1542 (1388-1713) | 2340 (1984-2758) | 4396 (3343-5772) |  |
|  |  | Female | 551 (492-618) | 794 (714-882) | 1207 (1023-1425) | 2280 (1729-3004) |  |
|  | 65 – 69 years | Male | 1740 (1554-1949) | 2499 (2251-2775) | 3783 (3212-4454) | 7061 (5387-9229) |  |
|  |  | Female | 900 (804-1009) | 1296 (1166-1440) | 1968 (1668-2320) | 3703 (2813-4867) |  |
|  | 70 – 74 years | Male | 2740 (2448-3067) | 3927 (3539-4357) | 5922 (5036-6958) | 10945 (8393-14211) |  |
|  |  | Female | 1532 (1368-1716) | 2202 (1982-2445) | 3335 (2830-3928) | 6237 (4754-8163) |  |
|  | 75 – 79 years | Male | 4446 (3976-4972) | 6348 (5728-7033) | 9510 (8111-11135) | 17283 (13367-22190) |  |
|  |  | Female | 2719 (2429-3043) | 3897 (3512-4323) | 5877 (4998-6905) | 10863 (8329-14106) |  |
|  | 80 – 84 years | Male | 7177 (6427-8010) | 10182 (9206-11255) | 15093 (12934-17575) | 26704 (20939-33688) |  |
|  |  | Female | 4911 (4392-5490) | 7004 (6322-7757) | 10474 (8941-12252) | 18948 (14690-24255) |  |
|  | ≥ 85 years | Male | 17408 (15683-19299) | 24102 (21966-26408) | 34307 (29931-39126) | 54970 (45303-65180) |  |
|  |  | Female | 15127 (13609-16796) | 21061 (19160-23123) | 30256 (26290-34667) | 49551 (40395-59535) |  |
| Values are 1-year death probabilities per 100,000 individuals. For this worked example, Austrian 2022/24 smoothed period life-table mortality was used as baseline mortality. Life-table mortality probabilities were converted to annual hazards, scaled by the pooled GOLD-specific hazard ratios, and converted back to 1-year mortality probabilities. Reported numbers equal p×100,000. 95% CIs were derived by applying the lower and upper 95% CI bounds of the hazard ratios; uncertainty in Austrian baseline mortality was not modelled. This example illustrates recalibration of the calculation framework to another national life table and does not validate country-specific COPD mortality in Austria. | | | | | | | |

Source: STATISTIK AUSTRIA, Geglättete Sterbetafel 2022–24 (Demographische Tafeln).

**e-Appendix methods:**

**Details on the search terms and data extraction process**

The data was collected in an Excel file, with a separate sheet for each GOLD grade. Documented information included first author’s last name, country, number of participants, follow-up duration, HRs with 95% CIs, reference group, covariate adjustment, definition of the reference group, and DOI. Data extraction was conducted by T.N. As an auxiliary quality-control step, a large language model (ChatGPT 5.2 Thinking, OpenAI) was used after human extraction and initial human judgments had been made. It was used to flag potential inconsistencies in extracted effect estimates, reference groups, adjustment variables, eligibility/exclusion decisions, and Newcastle-Ottawa Scale ratings. Model outputs were treated only as prompts for human re-checking. Suggested changes were accepted only when manual re-checking of the original publications confirmed that the original extraction or judgment should be changed. Ambiguous cases and any substantive changes to eligibility decisions, extracted estimates, exclusion rationales, or risk-of-bias judgments were discussed with R.H. Thus, the model was used to increase the chance of detecting overlooked issues, but all final decisions remained fully human and author-controlled.

**1-year mortality risks (probabilities) by sex, age, and GOLD grade in Germany**

To estimate age- and sex-specific mortality risks for COPD, period life table data (5-year age groups) were used. The life table probability of dying over the age interval, nq_x_, was first converted to an annual hazard to allow the application of hazard ratios. For each age group, the annual hazard was calculated as

h_x_=−ln(1−nq_x_)/n.

Grade-specific hazards were obtained as h_v_=HR_v_⋅h_x_ and converted to 1-year death probabilities p_v_=1−exp(−h_v_) (equivalently $p_{v}=1-{(1-p_{0})}^{{HR}_{v}}$ ). Reported values are shown as 100,000⋅p_v_ for readability. Accordingly, 95% confidence intervals (CIs) for grade-specific mortality risks were computed by multiplying the baseline hazard by the lower and upper 95% CI bounds of the HRs (uncertainty in the life-table baseline was not additionally modeled).

Because COPD is epidemiologically negligible at younger ages, analyses and reporting were restricted to age groups ≥45 years. The open-ended 85+ age group was aggregated from the components 85–89, 90–94, 95–99, and 100+. Component-specific annual hazards were calculated as above and then combined using a weighted average, with weights given by approximate person-years L_x_ ≈ n(l_x_−a_x_d_x_) (with a_x_=0.5). For the open-ended 100+ group, an assumption on the interval length was required (n=1 year for the open-ended 100+ group); a sensitivity analysis using an alternative assumption (e.g., n=5) showed only a minimal impact on the aggregated 85+ baseline (change <1%).

**Calculation of 95% confidence intervals for 1-year mortality risks**

The corresponding 95% confidence intervals for the mortality risks were calculated by using the lower and upper 95% confidence intervals of the grade-specific hazard ratios instead of the point estimates. Uncertainty in the life-table baseline was not propagated; accordingly, the reported confidence intervals reflect uncertainty in the hazard ratios only. The assumption is reasonable because mortality risks are known for the entire German population according to sex and 5-year age group (see e-Table 5).

**Supplementary references:**

1.	Mannino DM, Buist AS, Petty TL, Enright PL, Redd SC. Lung function and mortality in the United States: data from the First National Health and Nutrition Examination Survey follow up study. Thorax. 2003;58(5):388–93. doi: <https://doi.org/10.1136/thorax.58.5.388>.

2.	Ekberg-Aronsson M, Pehrsson K, Nilsson JA, Nilsson PM, Lofdahl CG. Mortality in GOLD stages of COPD and its dependence on symptoms of chronic bronchitis. Respir Res. 2005;6(1):98. doi: <https://doi.org/10.1186/1465-9921-6-98>.

3.	Mannino DM, Doherty DE, Sonia Buist A. Global Initiative on Obstructive Lung Disease (GOLD) classification of lung disease and mortality: findings from the Atherosclerosis Risk in Communities (ARIC) study. Respir Med. 2006;100(1):115–22. doi: <https://doi.org/10.1016/j.rmed.2005.03.035>.

4.	Stavem K, Sandvik L, Erikssen J. Can global initiative for Chronic Obstructive Lung Disease stage 0 provide prognostic information on long-term mortality in men? Chest. 2006;130(2):318–25. doi: <https://doi.org/10.1378/chest.130.2.318>.

5.	Lundback B, Eriksson B, Lindberg A, et al. A 20-year follow-up of a population study-based COPD cohort-report from the obstructive lung disease in Northern Sweden studies. COPD. 2009;6(4):263–71. doi: <https://doi.org/10.1080/15412550903061483>.

6.	Alfageme I, Reyes N, Merino M, et al. The effect of airflow limitation on the cause of death in patients with COPD. Chron Respir Dis. 2010;7(3):135–45. doi: <https://doi.org/10.1177/1479972310368692>.

7.	Mannino DM, Diaz-Guzman E, Buist S. Pre- and post-bronchodilator lung function as predictors of mortality in the Lung Health Study. Respir Res. 2011;12(1):136. doi: <https://doi.org/10.1186/1465-9921-12-136>.

8.	Mannino DM, Valvi D, Mullerova H, Tal-Singer R. Fibrinogen, COPD and mortality in a nationally representative U.S. cohort. COPD. 2012;9(4):359–66. doi: <https://doi.org/10.3109/15412555.2012.668249>.

9.	Johannessen A, Nilsen RM, Storebo M, Gulsvik A, Eagan T, Bakke P. Comparison of 2011 and 2007 Global Initiative for Chronic Obstructive Lung Disease guidelines for predicting mortality and hospitalization. Am J Respir Crit Care Med. 2013;188(1):51–9. doi: <https://doi.org/10.1164/rccm.201212-2276OC>.

10.	Leivseth L, Brumpton BM, Nilsen TI, Mai XM, Johnsen R, Langhammer A. GOLD classifications and mortality in chronic obstructive pulmonary disease: the HUNT Study, Norway. Thorax. 2013;68(10):914–21. doi: <https://doi.org/10.1136/thoraxjnl-2013-203270>.

11.	Mattila T, Vasankari T, Kanervisto M, et al. Association between all-cause and cause-specific mortality and the GOLD stages 1-4: A 30-year follow-up among Finnish adults. Respir Med. 2015;109(8):1012–8. doi: <https://doi.org/10.1016/j.rmed.2015.06.002>.

12.	Brusse-Keizer M, Klatte M, Zuur-Telgen M, Koehorst-Ter Huurne K, van der Palen J, VanderValk P. Comparing the 2007 and 2011 GOLD Classifications as Predictors of all-Cause Mortality and Morbidity in COPD. COPD. 2017;14(1):7–14. doi: <https://doi.org/10.1080/15412555.2016.1206875>.

13.	Gedebjerg A, Szepligeti SK, Wackerhausen LH, et al. Prediction of mortality in patients with chronic obstructive pulmonary disease with the new Global Initiative for Chronic Obstructive Lung Disease 2017 classification: a cohort study. Lancet Respir Med. 2018;6(3):204–12. doi: <https://doi.org/10.1016/S2213-2600(18)30002-X>.

14.	Perez-Padilla R, Wehrmeister FC, de Oca MM, et al. Outcomes for symptomatic non-obstructed individuals and individuals with mild (GOLD stage 1) COPD in a population based cohort. Int J Chron Obstruct Pulmon Dis. 2018;13:3549–61. doi: <https://doi.org/10.2147/copd.S175527>.

15.	Wijnant SRA, De Roos E, Kavousi M, et al. Trajectory and mortality of preserved ratio impaired spirometry: the Rotterdam Study. Eur Respir J. 2020;55(1). doi: <https://doi.org/10.1183/13993003.01217-2019>.

16.	Brat K, Svoboda M, Zatloukal J, et al. The Relation Between Clinical Phenotypes, GOLD Groups/Stages and Mortality in COPD Patients - A Prospective Multicenter Study. Int J Chron Obstruct Pulmon Dis. 2021;16:1171–82. doi: <https://doi.org/10.2147/copd.S297087>.

17.	Damkjaer M, Hakansson K, Kallemose T, Ulrik CS, Godtfredsen N. Statins in High-Risk Chronic Obstructive Pulmonary Disease Outpatients: No Impact on Time to First Exacerbation and All-Cause Mortality - The STATUETTE Cohort Study. Int J Chron Obstruct Pulmon Dis. 2021;16:579–89. doi: <https://doi.org/10.2147/COPD.S296472>.

18.	Guo C, Yu T, Chang LY, et al. Mortality risk attributable to classification of chronic obstructive pulmonary disease and reduced lung function: A 21-year longitudinal cohort study. Respir Med. 2021;184:106471. doi: <https://doi.org/10.1016/j.rmed.2021.106471>.

19.	Hasenstab KA, Yuan N, Retson T, et al. Automated CT Staging of Chronic Obstructive Pulmonary Disease Severity for Predicting Disease Progression and Mortality with a Deep Learning Convolutional Neural Network. Radiol Cardiothorac Imaging. 2021;3(2):e200477. doi: <https://doi.org/10.1148/ryct.2021200477>.

20.	He D, Sun Y, Gao M, et al. Different Risks of Mortality and Longitudinal Transition Trajectories in New Potential Subtypes of the Preserved Ratio Impaired Spirometry: Evidence From the English Longitudinal Study of Aging. Front Med (Lausanne). 2021;8:755855. doi: <https://doi.org/10.3389/fmed.2021.755855>.

21.	Washio Y, Sakata S, Fukuyama S, et al. Risks of Mortality and Airflow Limitation in Japanese Individuals with Preserved Ratio Impaired Spirometry. Am J Respir Crit Care Med. 2022;206(5):563–72. doi: <https://doi.org/10.1164/rccm.202110-2302OC>.

22.	Kaaks R, Christodoulou E, Motsch E, et al. Lung function impairment in the German Lung Cancer Screening Intervention Study (LUSI): prevalence, symptoms, and associations with lung cancer risk, tumor histology and all-cause mortality. Transl Lung Cancer Res. 2022;11(9):1896–911. doi: <https://doi.org/10.21037/tlcr-22-63>.

23.	Cheng W, Zhou A, Zeng Y, et al. Prediction of Hospitalization and Mortality in Patients with Chronic Obstructive Pulmonary Disease with the New Global Initiative for Chronic Obstructive Lung Disease 2023 Group Classification: A Prospective Cohort and a Retrospective Analysis. Int J Chron Obstruct Pulmon Dis. 2023;18:2341–52. doi: <https://doi.org/10.2147/COPD.S429104>.

24.	Cadham CJ, Oh H, Han MK, et al. The prevalence and mortality risks of PRISm and COPD in the United States from NHANES 2007-2012. Respir Res. 2024;25(1):208. doi: <https://doi.org/10.1186/s12931-024-02841-y>.

25.	Whittaker H, Rothnie KJ, Quint JK. Cause-specific mortality in COPD subpopulations: a cohort study of 339 647 people in England. Thorax. 2024;79(3):202–8. doi: <https://doi.org/10.1136/thorax-2022-219320>.

26.	Egerod L, Ter Haar E, Karsdal MA, et al. Comorbidity profiles in chronic obstructive pulmonary disease: a multicohort study. ERJ Open Res. 2025;11(5). doi: <https://doi.org/10.1183/23120541.01289-2024>.

27.	Hyung K, Lee HW, Choi JY, et al. Impact of the Race-Neutral Equation on Mortality Risk Prediction in Korean Patients With Chronic Obstructive Pulmonary Disease. Respirology. 2025;30(9):831–9. doi: <https://doi.org/10.1111/resp.70052>.

28.	Ke Y, Zhao Y, Sun D, et al. All-cause and cause-specific mortality in individuals with COPD in China: a 16-year follow-up cohort study. Eur J Epidemiol. 2025;40(6):681–91. doi: <https://doi.org/10.1007/s10654-025-01252-7>.

29.	Lin CH, Li YR, Cheng SL, et al. Prognostic risk profiling in COPD using Global Initiative for Chronic Obstructive Lung Disease 2023 ABE and comorbidity assessment: evidence from a register-based COPD cohort. J Glob Health. 2025;15:04152. doi: <https://doi.org/10.7189/jogh.15.04152>.

30.	Ma J, Shen C, Jia Y, et al. Independent and joint relationships of physical activity and pulmonary function with the risk of all-cause mortality. BMJ Open Respir Res. 2025;12(1). doi: <https://doi.org/10.1136/bmjresp-2024-002818>.

31.	Niedermaier T, Alter P, Jorres R, Vogelmeier C, Holle R. Parametric estimation of age-, sex-, and spirometry grade-specific mortality in a cohort of COPD patients from Germany: Results from COSYCONET. Respir Med. 2025;247:108280. doi: <https://doi.org/10.1016/j.rmed.2025.108280>.

32.	Ogata H, Tsubouchi K, Takano T, et al. Mortality and exacerbation risk according to GOLD and STAR severity stages of COPD: a 5-year multicenter prospective cohort study. Sci Rep. 2025;15(1):19097. doi: <https://doi.org/10.1038/s41598-025-05033-w>.

33.	Wallstrom O, Stridsman C, Backman H, et al. Risk of exacerbations, hospitalisation, and mortality in adults with physician-diagnosed chronic obstructive pulmonary disease with normal spirometry and adults with preserved ratio impaired spirometry in Sweden: retrospective analysis of data from a nationwide cohort study. Lancet Reg Health Eur. 2025;54:101322. doi: <https://doi.org/10.1016/j.lanepe.2025.101322>.

34.	Casanova C, Gonzalez-Davila E, de Torres JP, et al. Lung Diffusing Capacity Improves the Prognostic Validity of the GOLD Spirometric Staging in COPD. Arch Bronconeumol. 2026;62(1):20–7. doi: <https://doi.org/10.1016/j.arbres.2025.05.004>.

35.	Alupo P, Wosu AC, Mahofa A, et al. Incidence and predictors of COPD mortality in Uganda: A 2-year prospective cohort study. PLoS One. 2021;16(2):e0246850. doi: <https://doi.org/10.1371/journal.pone.0246850>.

36.	Athlin A, Giezeman M, Hasselgren M, et al. Prediction of Mortality Using Different COPD Risk Assessments - A 12-Year Follow-Up. Int J Chron Obstruct Pulmon Dis. 2021;16:665–75. doi: <https://doi.org/10.2147/COPD.S282694>.

37.	Backman H, Vanfleteren L, Mannino DM, Ekstrom M. Severity of Airflow Obstruction Based on FEV(1)/FVC Versus FEV(1) Percent Predicted in the General U.S. Population. Am J Respir Crit Care Med. 2024;210(11):1308–16. doi: <https://doi.org/10.1164/rccm.202310-1773OC>.

38.	Bavishi S, Chaudhary D, Li J, Naik S, Abedi V, Zand R. Long-term mortality in ischemic stroke patients with concomitant chronic obstructive pulmonary disease. J Stroke Cerebrovasc Dis. 2022;31(11):106701. doi: <https://doi.org/10.1016/j.jstrokecerebrovasdis.2022.106701>.

39.	Bertels X, Riemann S, Vauterin D, Lahousse L, Brusselle GG. All-Cause Mortality of Staging of Airflow Obstruction by Ratio-categorized Patients with Chronic Obstructive Pulmonary Disease among the General Population. Am J Respir Crit Care Med. 2024;210(11):1374–6. doi: <https://doi.org/10.1164/rccm.202311-2144LE>.

40.	Bhatt SP, Nakhmani A, Fortis S, et al. FEV(1)/FVC Severity Stages for Chronic Obstructive Pulmonary Disease. Am J Respir Crit Care Med. 2023;208(6):676–84. doi: <https://doi.org/10.1164/rccm.202303-0450OC>.

41.	Bhatta L, Leivseth L, Mai XM, et al. GOLD Classifications, COPD Hospitalization, and All-Cause Mortality in Chronic Obstructive Pulmonary Disease: The HUNT Study. Int J Chron Obstruct Pulmon Dis. 2020;15:225–33. doi: <https://doi.org/10.2147/copd.S228958>.

42.	Borvik T, Braekkan SK, Enga K, et al. COPD and risk of venous thromboembolism and mortality in a general population. Eur Respir J. 2016;47(2):473–81. doi: <https://doi.org/10.1183/13993003.00402-2015>.

43.	Borvik T, Braekkan SK, Evensen LH, et al. Chronic Obstructive Pulmonary Disease and Risk of Mortality in Patients with Venous Thromboembolism-The Tromso Study. Thromb Haemost. 2020;120(3):477–83. doi: <https://doi.org/10.1055/s-0039-3400744>.

44.	Chan HP, Mukhopadhyay A, Chong PL, et al. Prognostic utility of the 2011 GOLD classification and other multidimensional tools in Asian COPD patients: a prospective cohort study. Int J Chron Obstruct Pulmon Dis. 2016;11:823–9. doi: <https://doi.org/10.2147/COPD.S96790>.

45.	Chen CZ, Ou CY, Hsu CH, Hsiue TR. Validation of the GOLD 2013 classification in predicting exacerbations and mortality in Taiwanese patients with chronic obstructive pulmonary disease. J Formos Med Assoc. 2015;114(12):1258–66. doi: <https://doi.org/10.1016/j.jfma.2014.12.003>.

46.	Dusser D, Wise RA, Dahl R, et al. Differences in outcomes between GOLD groups in patients with COPD in the TIOSPIR((R)) trial. Int J Chron Obstruct Pulmon Dis. 2016;11:133–45. doi: <https://doi.org/10.2147/COPD.S97924>.

47.	Garcia-Aymerich J, Serra Pons I, Mannino DM, Maas AK, Miller DP, Davis KJ. Lung function impairment, COPD hospitalisations and subsequent mortality. Thorax. 2011;66(7):585–90. doi: <https://doi.org/10.1136/thx.2010.152876>.

48.	Holmgaard DB, Mygind LH, Titlestad IL, et al. Serum vitamin D in patients with chronic obstructive lung disease does not correlate with mortality--results from a 10-year prospective cohort study. PLoS One. 2013;8(1):e53670. doi: <https://doi.org/10.1371/journal.pone.0053670>.

49.	Holmgaard DB, Mygind LH, Titlestad IL, et al. Plasma YKL-40 and all-cause mortality in patients with chronic obstructive pulmonary disease. BMC Pulm Med. 2013;13:77. doi: <https://doi.org/10.1186/1471-2466-13-77>.

50.	Hwang HJ, Lee SM, Seo JB, et al. Quantitative Vertebral Bone Density Seen on Chest CT in Chronic Obstructive Pulmonary Disease Patients: Association with Mortality in the Korean Obstructive Lung Disease Cohort. Korean J Radiol. 2020;21(7):880–90. doi: <https://doi.org/10.3348/kjr.2019.0551>.

51.	Lee HM, Liu M, Lee K, Luo Y, Wong ND. Does low vitamin D amplify the association of COPD with total and cardiovascular disease mortality? Clin Cardiol. 2014;37(8):473–8. doi: <https://doi.org/10.1002/clc.22284>.

52.	Nishimura K, Kusunose M, Shibayama A, Nakayasu K. Comparison of Disease Severity Classifications of Chronic Obstructive Pulmonary Disease: GOLD vs. STAR in Clinical Practice. Diagnostics (Basel). 2024;14(6). doi: <https://doi.org/10.3390/diagnostics14060646>.

53.	Pedone C, Giua R, Scichilone N, Bellia V, Antonelli-Incalzi R. GOLD Staging System is Appropriate to Predict Mortality in Older People With Chronic Obstructive Pulmonary Disease. Arch Bronconeumol (Engl Ed). 2018. doi: <https://doi.org/10.1016/j.arbres.2018.01.022>.

54.	Shavelle RM, Paculdo DR, Kush SJ, Mannino DM, Strauss DJ. Life expectancy and years of life lost in chronic obstructive pulmonary disease: findings from the NHANES III Follow-up Study. Int J Chron Obstruct Pulmon Dis. 2009;4:137–48. doi: <https://doi.org/10.2147/copd.s5237>.

55.	Song Q, Lin L, Cheng W, et al. Clinical-functional characteristics and risk of exacerbation and mortality among more symptomatic patients with chronic obstructive pulmonary disease: a retrospective cohort study. BMJ Open. 2023;13(3):e065625. doi: <https://doi.org/10.1136/bmjopen-2022-065625>.

56.	Soriano JB, Alfageme I, Almagro P, et al. Distribution and prognostic validity of the new Global Initiative for Chronic Obstructive Lung Disease grading classification. Chest. 2013;143(3):694–702. doi: <https://doi.org/10.1378/chest.12-1053>.

57.	Soriano JB, Lamprecht B, Ramirez AS, et al. Mortality prediction in chronic obstructive pulmonary disease comparing the GOLD 2007 and 2011 staging systems: a pooled analysis of individual patient data. Lancet Respir Med. 2015;3(6):443–50. doi: <https://doi.org/10.1016/S2213-2600(15)00157-5>.

58.	Valvi D, Mannino DM, Mullerova H, Tal-Singer R. Fibrinogen, chronic obstructive pulmonary disease (COPD) and outcomes in two United States cohorts. Int J Chron Obstruct Pulmon Dis. 2012;7:173–82. doi: <https://doi.org/10.2147/COPD.S29892>.

59.	Yong SH, Leem AY, Kim YS, et al. Hepatic Fibrosis Assessed Using Fibrosis-4 Index Is Predictive of All-Cause Mortality in Patients with Chronic Obstructive Pulmonary Disease. Int J Chron Obstruct Pulmon Dis. 2020;15:831–9. doi: <https://doi.org/10.2147/COPD.S242863>.

60.	Zou W, Ou J, Wu F, et al. Risk of All-Cause Mortality in Mild Chronic Obstructive Pulmonary Disease: Evidence From the NHANES III and 2007-2012. Int J Chron Obstruct Pulmon Dis. 2025;20:217–29. doi: <https://doi.org/10.2147/COPD.S497634>.
